# TRPA1 exacerbates selective retinal ganglion cell vulnerability under acute ocular hypertension

**DOI:** 10.1186/s40478-025-01974-5

**Published:** 2025-04-05

**Authors:** Wenhan Lu, Yu Wang, Wei Hu, Xinyi Lin, Xiaoyu Tong, Yi Tian, Yuning Chen, Yicong Wang, Yan Xiao, Hongfang Yang, Yi Feng, Xinghuai Sun

**Affiliations:** 1 Department of Ophthalmology & Visual Science, Eye & ENT Hospital, Shanghai Medical College, Fudan University, Shanghai, 200031 China; 2 State Key Laboratory of Medical Neurobiology and MOE Frontiers Center for Brain Science, Institutes of Brain Science, Fudan University, Shanghai, 200032 China; 3 NHC Key Laboratory of Myopia and Related Eye Diseases, Shanghai Key Laboratory of Visual Impairment and Restoration, Chinese Academy of Medical Sciences, Fudan University, Shanghai, 200031 China; 4 Department of Integrative Medicine and Neurobiology, School of Basic Medical Sciences, State Key Laboratory of Medical Neurobiology and MOE Frontiers Center for Brain Science, Institutes of Brain Science, Fudan University, Shanghai, 200032 China; 5 Shanghai Key Laboratory of Acupuncture Mechanism and Acupoint Function, Shanghai, 200032 China; 6 Department of Oncology, Shanghai Medical College, Fudan University, Shanghai, 200032 China; 7 Reproductive Medicine Center, Zhongshan Hospital, Shanghai Medical College, Fudan University, Shanghai, 200040 China

**Keywords:** TRPA1, Acute ocular hypertension, RGC central projection, Neuronal selective vulnerability, Tissue optical clearing

## Abstract

Acute ocular hypertension (AOH), a major cause of progressive irreversible vision loss, showed significant retinal ganglion cell (RGC) degeneration as well as selective RGC vulnerability upon functional tests, yet the underlyding mechanisms remain incompletely understood. Here, we report the activation of transient receptor potential ankyrin 1 (TRPA1), a mechanosensitive ion channel on RGCs under AOH by RT-qPCR, Western blot, immunofluorescent, flow cytometry and calcium imaging tests. Downstream CaMKII/CREB pathways were evaluated, showing significantly elevated phospho-CaMKII and down-regulated phospho-CREB1 under AOH. Further, by applying a modified whole-brain clearing method, the region-specific RGC axonal damage among lateral geniculate nuclei (LGN) subregions were adopted to detect the involvement of TRPA1 on selective RGC vulnerability. Together with tissue-specific knock-out or channel inhibition test, the exacerbation of TRPA1 on RGC degeneration as well as selective injury tendency under AOH was confirmed. In virtue of our modified whole-brain clearing method, our data confirmed the innovational method to study the mechanisms behind selective vulnerability of neuronal cells, and in the meantime revealed the potential therapeutic opportunity of targeting TRPA1 for patients suffering from AOH attack.

**Supplementary Information:**

The online version contains supplementary material available at 10.1186/s40478-025-01974-5.

## Introduction

Acute ocular hypertension (AOH), characterized by a sudden intraocular pressure (IOP) elevation, occurs commonly in clinical scenarios including acute glaucomatous attack, intraoperative ocular hypertension due to high perfusion pressure, or transient postoperative IOP spike, posing a significant threat to intraocular structures, particularly the retina. In the past years, population-based studies are showing a three-fold increased risk of severe, bilateral visual impairment of primary angle-closure glaucoma (PACG) compared with primary open-angle glaucoma (POAG), while acute PACG, which is characterized by a sudden and dramatic rise in IOP, expects the highest risk of blindness among different types of glaucoma [1].

To date, various mechanisms involved in retinal ganglion cell (RGC) degeneration under AOH have been discovered, together with emerging potential therapeutic strategies. Although recent findings revealed axonal ischemia [2, 3], oxidative stress as well as mitochondrial dysfunction [4], glial cell dysfunction [5], extracellular matrix (ECM) remodeling [6], etc., all lead to progressive neurodegeneration in glaucoma, the mechanical stress imposed on the posterior structures of the eye, particularly at the optic nerve head (ONH), is still the most significant mechanism as well as currently the only modifiable risk factor with clinical benefits [7]. Cumulative evidences reveal that RGCs experience multiple programmed cell death pathways including apoptosis, necroptosis, pyroptosis and ferroptosis triggered by activation of various cellular or ECM responders-right during the pressure elevation, yet continue after the IOP is well-controlled [8–10]. However, there were still unmet clinical needs as only a few drugs, when targeting certain pathways, showed protective effects on RGCs upon IOP elevation. The urge for uncovering the mechanisms underlying the clinically-found progressive vision loss after acute elevation of IOP cohere with the aim of investigating novel therapeutic options for patients suffering from glaucomatous neurodegeneration.

Being inspired by the mechanical stress IOP elevation imposed on the eye structures, we paid attention to the transient receptor potential (TRP) ion channels, one of the most important sensory-transducing ion channel families. TRP is a group of evolutionarily conserved cationic ion channels that respond to chemical, physical, metabolic and ionic stimuli [11], therefore being called mechanicoceptors, thermoreceptors and nociceptors. TRPs express and function in the glia, photoreceptors, bipolar cells, horizontal and amacrine cells as well as RGCs to modulate pathophysiological responses to various stimuli. Though scientists have explored more on the role of transient receptor potential vanilloid 4 (TRPV4), TRPV1, transient receptor potential melastatin 1 (TRPM1) in RGC pathophysiology, TRPA1, a major subfamily of TRP, becomes an attractive target in terms of their presence in sensory neurons, e.g., the DRG, trigeminal, and nodose ganglia at the tissue level, probably mediating pain [12, 13] (known as nociceptors), thermal sensation [14] (known as thermoreceptors) and neuroinflammation [15, 16]. Like other TRP members, TRPA1 channels are involved in the perception of mechanical stimuli [17], which hinted its possible participation in stretch-related ocular diseases-the most well-known one being IOP elevation. Retina was previously studied [18] and showed TRPA1 mediating retinal ischemia and ischemia-reperfusion damage in mice [19]. It was also found that when TRPA1 was activated, dissociated adult RGCs would experience larger calcium influx elevations that are comparable to the effects of saturating concentrations of glutamate [11]. Questions remained on how these channels respond to mechanical stretch or press in cases of elevation of intraocular pressure (IOP), the downstream mechanisms, and if they contribute to the selective RGC vulnerability under pathological conditions like AOH [19].

Here, by RT-qPCR screening of main TRP family members, we found TRPA1 with distinct upregulation in the inner retina under AOH, as well as the elevation of Ca^2+^ influx in RGCs when activated. These findings were confirmed by RGC-specific knock-out of *Trpa1*, along with the protective TRPA1 antagonist against AOH-induced RGC degeneration. We also revealed the downstream Ca(2+)/CaMKII/CREB pathway through which the RGCs degenerate upon TRPA1 activation. Then, taking advantage of our recently-developed new approach of evaluating the RGC central projection changes as well as the selective vulnerability of different RGC groups [20], we were able to explore TRPA1’s role on the possible mechanisms behind AOH-induced RGC injury and selective vulnerability. With these, we provided potential individualized therapeutic opportunity for neuronal protection in patients suffering from IOP elevation.

## Materials and methods

### Mice

*Trpa1*^*fl/fl*^ and *Thy1-Cre* transgenic mice were obtained from Cyagen Biosciences Inc., Guangzhou, China (#S-CKO-10065 and #C001306, respectively), and C57BL/6J mice were purchased from the Shanghai Laboratory Animal Center (SLAC), Shanghai, China. *Trpa1*^*fl/fl*^ and *Thy1-Cre* transgenic mice were genotyped prior to being used for experiments, using primers recommended by the vendor (Additional File 1: Fig. S2). For *Trpa1*^*fl/fl*^, the mutant forward primer was GAG AGT CCT GTA TTG CCT AGC TG and the reverse primer was AGA AGT GCC CTT TCA AGA ACA GAA. For *Thy1-Cre*, the region1 forward primer was TAG CTT TCC CCA CCA CAG AATC and the reverse primer was GAC GAT GAA GCA TGT TTA GCT GGC, the region2 forward primer was CTA TCA GGG ATA CTC CTC TTT GCC and the reverse primer was GAT ACA GGA ATG ACA AGC TCA TGGT. DNA gel electrophoresis of *Trpa1*^*fl/fl*^ animals showing a single band at 201 bp indicated successful gene mutation, whereas wild type animals showed a single band at 134 bp. A single band at 398 bp for Cre region1 indicated carrying *Thy1-Cre.* Each animal was genetically verified before use. All of these mice were housed in a barrier facility with a 12 h light/12 h dark cycle, humidity of 34–40% and temperature at 21 ~ 23 °C. Mice were fed ad libitum with the same standard laboratory chow (SLAC, Shanghai, China) and given free access to water.

### Acute ocular hypertension model

A well-acknowledged intracameral irrigation model was adopted for AOH model construction as previously reported [20]. In brief, mice were anesthetized with avertin (6.75 mg/mL) at a dose of 20 µL/g body weight (half of standard dose), which last approximately 50 min in average. A second dose of avertin at 10 µL/g body weight would be administered with care when needed once animals showed any sign of recovery. A mixture of 0.5% tropicamide and 0.5% phenylephrine eye drops was used to dilate the pupil. Paracentesis of anterior chamber was performed with a 32-gauge sterile needle connected to a saline reservoir at a height of 97 cm, raising the IOP to 70 mmHg. During the process, IOP was measured with Tonolab (Icare, Finland) every 10 min. 80 min later, the needle was gently removed. The contralateral left eye was only cannulated and served as control in this study.

### Open field test

**Fig. 1:**
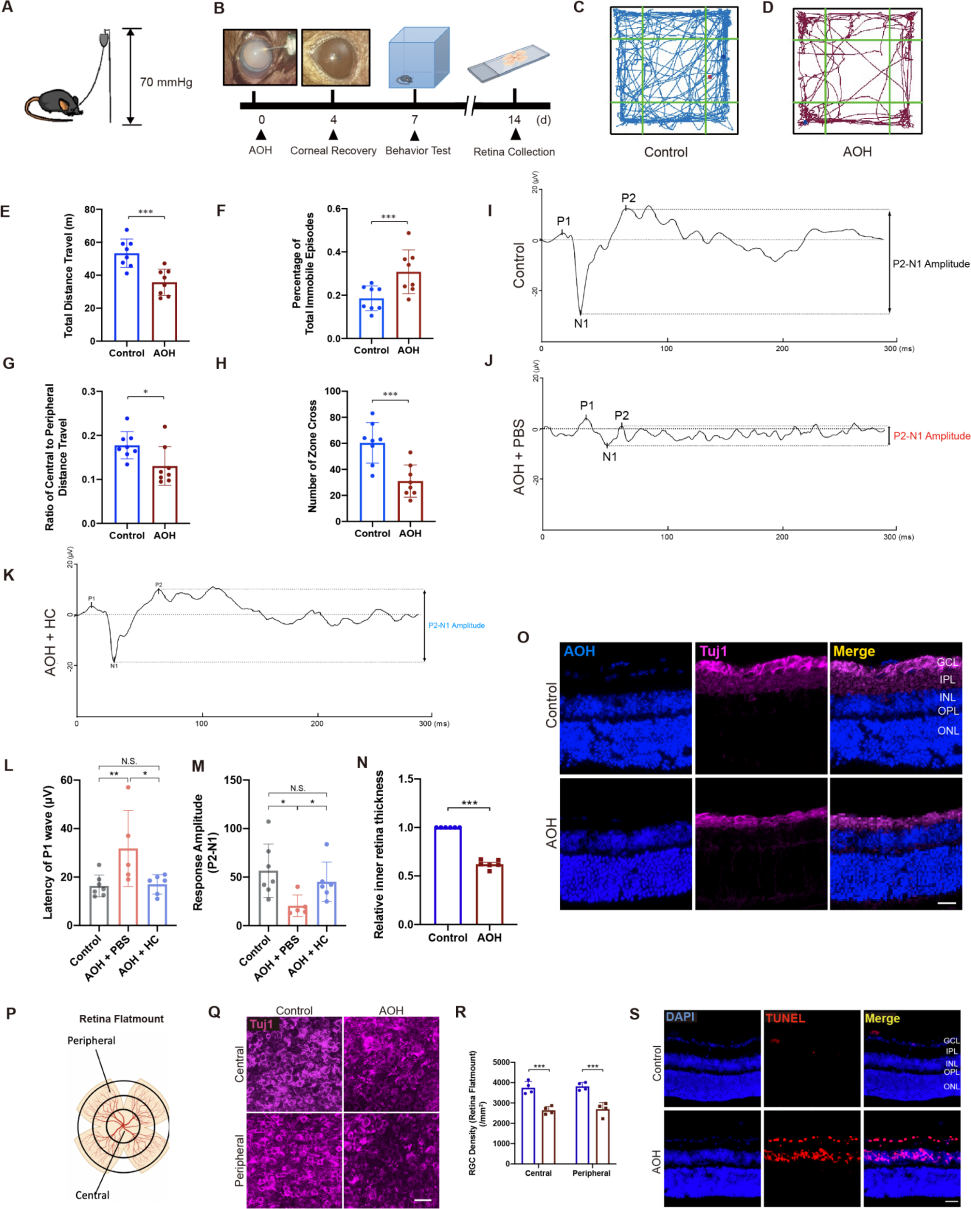
AOH Model construction and RGC damage under AOH. **(A)** Schematic illustration of AOH model construction. **(B)** Experimental design. **(C-D)** Track plots of **(C)** control and **(D)** 7-day AOH mice in the open field. **(E-H)** Comparison of **(E)** total distance travelled, **(F)** percentage of total immobile episodes, **(G)** ratio of central to peripheral distance travelled and **(H)** number of zone cross between AOH and control mice (*n* = 8 for each group, unpaired *t-test*, *P < 0.05, ***P < 0.01). **(I)** F-VEP detection waveforms of control mice, **(J)** AOH mice with intravitreal injection of PBS and **(K)** AOH mice with intravitreal injection of HC-030031 (HC). **(L)** Quantitative analysis of the latency of P1 (*n* = 7 for control mice, *n* = 5 for AOH + PBS mice and *n* = 6 for AOH + HC mice) and **(M)** amplitude of P2-N1. **(N)** Statistical analysis of relative inner retinal thickness (GCL + IPL + INL) (*n* = 6 for each experiment, paired *t-test*, **P < 0.02, ***P < 0.01). **(O)** Immunofluorescent staining of retinal slices. Scale bar: 25 μm. **(P)** Graphic illustration indicating the central and peripheral retina. **(Q)** Immunofluorescent staining of retinal flatmount showing RGC counts at both the center and the periphery (*n* = 4 for each group, paired *t-test*, **P < 0.02). **(R)** Statistical analysis of RGC counts at both the center and periphery (*n* = 4 for each experiment, paired *t-test*, ***P < 0.01). **(S)** TUNEL staining of retinal slices showing cell apoptosis among GCL and INL 7 days after AOH. Scale bar: 25 μm. Results are presented as mean ± standard deviation (SD). AOH, acute ocular hypertension; GCL, ganglion cell layer; INL, inner nuclear layer; IPL, inner plexiform layer; LGN, lateral geniculate nucleus; OC, optic chiasm; ONL, outer nuclear layer; OPL, outer plexiform layer; OT, optic tract; SC, superior colliculi

The influence of vision damage on the behavior of mice was assessed in the open field arena as previously reported [21]. In brief, 50 cm (L) × 50 cm (W) × 50 cm (H) white polyvinyl chloride (PVC) open field test arena was prepared. The whole arena was divided into peripheral and central zones, with the latter locating at the center with dimensions of 25 cm (L) × 25 cm (W) (Fig. 1C-D). Test was conducted in the morning from 9:00 till 12:00 am, when mice were placed in the middle of the open field arena and were allowed to explore for 10 min. The parameters that were observed and analyzed using Supermaze Video Tracking Software, version 3.3.0.0 (Shanghai Xinsoft Information Technology Co., LTD, China) included total distance travelled, central/peripheral distance travelled, duration of immobile episodes and number of zone crossing. In each study group, at least 5 to 8 individuals were included. Healthy mice were set as control for AOH behavior test, while in *Trpa1* knock-out or HC antagonist test, those unilateral AOH mice with RGCs expressing TRPA1 or intravitreously given PBS were set as control groups. One-way ANOVA and unpaired *t-test* was applied for statistical analysis. All behaviors were assessed 1 week after AOH modeling.

### Flash visual-evoked potential examination

Flash visual-evoked potential (F-VEP) detection was performed following previously-reported protocols [22]. Briefly, the control mice or 14-day AOH models with PBS or HC treatment were dark-adapted for at least 8 h and then anesthetized with avertin at a dose of 20 µL/g, and a mixture of 0.5% tropicamide and 0.5% phenylephrine eye drops was used to dilate the pupil. Mice were then fixed, with the recording and reference electrodes placed under the scalp at the occipital tuberosity and under the skin of the nose, respectively. After placing the ground electrode at the tail, an opaque black blindfold was used to cover the left eye, and the mice went through examination of F-VEP (Espion E3; Diagnosys UK) with stimulation flash of 3 cd s^− 1^ m^− 2^ light intensity and 1.02 Hz frequency. The F-VEP inspections were carried out with at least three consecutive measurements performed each time. In each study group, at least 5 to 7 individuals were included. Healthy mice were set as control for AOH group, while those AOH mice with intravitreous injection of PBS were also the control for HC treatment group. One-way ANOVA and unpaired *t-test* was applied for statistical analysis.

### Flow cytometry analysis

The flow cytometry analysis was conducted 2 weeks after AOH modeling. The eye contralateral to AOH eye from the same individual was set as the control, i.e., a self-control. The retinas obtained from the anesthetized mice were digested in the enzyme buffer containing 5 mg/mL papain (Sigma, Germany), 1 mg/mL DNase I (Thermo Fisher, USA) and 1% FBS in PBS for 30 min at 37 °C. Retinal cells were carefully dissociated and filtered through a 70 μm cell strainer (Beyotime, China). After washing with the Cell Staining Buffer (#420201, Biolegend, USA), cell suspension was centrifuged at 500 g for 5 min, and then the pellet was re-suspended in 500 µL stain buffer. For flow cytometry analysis of TRPA1 expression, cells were stained for viability and blocked, then incubated for 30 min on ice in buffer containing phycoerythrin (PE)-conjugated antibody for Thy1.1 (#109006, Biolegend, USA) and Alexa-647 conjugated TRPA1 antibody (NB110-40763AF647, Novus, USA). The suspension was then washed twice. RGC carrying PE or Alexa-647 signal can be detected by BD fluorescence-activated cell sorting (FACS) Verse Flow Cytometer (BD, USA) and analyzed by FlowJo V10.8.1 software (TreeStar, USA). Paired *t-test* was applied for statistical analysis, with the relative values of AOH eye calculated as divided by the values of the control eye.

### Real-time quantitative PCR analysis

For real-time quantitative PCR analysis, the eye contralateral to AOH eye from the same individual was set as the control. The whole retinae were collected in time sequence at day3, day5, day7 and day14-post AOH modeling. Total RNA of retina was isolated using Trizol reagent (9109Q, Takara Bio, Inc. Japan) according to the manufacturer’s instructions, and single-stranded cDNA was synthesized from each sample (2 µg) with PrimeScript RT Master Mix (#RR036A, Takara Bio, Inc., Japan). Real-time quantitative PCR (RT-qPCR) was performed with an ABI PRISM 7300Plus sequence detection system (Applied Biosystems, Foster City, CA). The PCR parameters were set according to the manufacturer’s protocols, and amplifications were performed with a SYBR Premix Ex Taq kit (#RR420A, Takara Bio, Inc., Japan). For each sample, duplicate reactions were performed in 96-well plates, and all primers were checked to ensure the uniformity of the target gene. Primer quality was further demonstrated by the dissociation curve in the RT-qPCR test prior to use. The primer sequences were listed in Table S1. Relative gene expression was determined with the 2^–∆∆CT^ formula. At least three independent experiments were performed. Paired *t-test* was applied for statistical analysis.

**Fig. 2:**
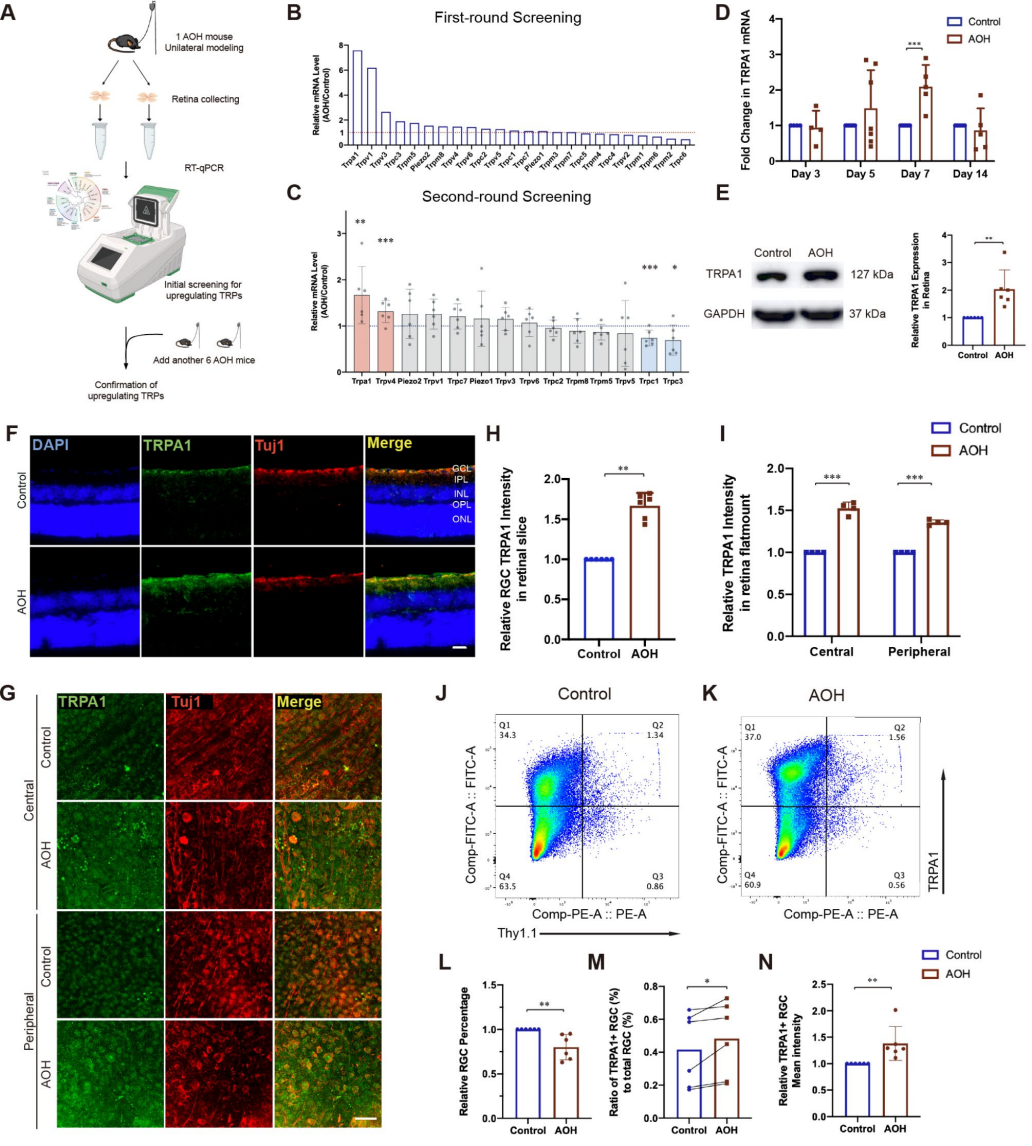
Retinal damage and TRPA1 change after AOH. (**A**) Schematic illustration of workflow for screening of relative expression of all the genes encoding TRP and PIEZO channel families in AOH retina. **(B)** Results for first-round screening and **(C)** results for second-round screening. The relative mRNA level was calculated as the fold change of respective genes in AOH retina compared with control retina (*n* = 6 for each experiment, paired *t-test*, *P < 0.05, **P < 0.02, ***P < 0.01). **(D)** Relative expression of TRPA1 in time sequence by RT-qPCR. At least three independent experiments were performed in duplicates (***P < 0.01). **(E)** Gel blot and densitometry analysis of TRPA1 showed TRPA1 upregulation in AOH (*n* = 6, paired *t-test*, **P < 0.02). **(F-G)** Expression pattern of TRPA1 in **(F)** retina slice and in **(G)** central and peripheral retinal flatmounts under physiological or AOH conditions. Statistical analysis of relative intensity of TRPA1 in GCL were shown in **(H)** and **(I)**, respectively. Scale bar: 25 μm. **(J-N)** Flow cytometry analysis showing **(L)** decreased RGC proportion under AOH (*n* = 6, paired *t-test*, **P < 0.02) with **(M)** elevated TRPA1^+^ RGC percentage (*n* = 6, paired *t-test*, *P < 0.05) and **(N)** average TRPA1 expression indicated by FITC intensity (*n* = 6, paired *t-test*, **P < 0.02). Live Retinal cells and single cells were sorted, and cross gates divided all retinal cells in to TRPA1^+^ RGCs (Q2), TRPA1^−^ RGCs (Q3), TRPA1^+^ retinal cells other than RGCs (Q1) and TRPA1^−^ retinal cells other than RGCs (Q4). Results are presented as mean ± standard deviation (SD). AOH, acute ocular hypertension; GCL, ganglion cell layer; INL, inner nuclear layer; IPL, inner plexiform layer; OPL, outer plexiform layer; ONL, outer nuclear layer

For identification of TRP and PIEZO channel families’ expression in AOH retina compared with control retina, a stepwise RT-qPCR screening was applied. In brief, a first-round screening for 1 individual was applied for primary selection of TRPs and PIEZO expression in retina. Up-regulated genes were further confirmed with a second-round screening, where another 6 individuals were invited (Fig. 2A). The relative mRNA level was calculated as the fold change of respective genes in AOH retina compared with control retina. Paired *t-test* was applied for statistical analysis.

### Western blot analysis

For western blot analysis of protein level, the eye contralateral to AOH eye from the same individual was set as the control. In *Trpa1* knock-out test, those unilateral AOH mice with RGCs expressing TRPA1 were set as control group. The whole retinae were collected on day14 after AOH modeling for detection of protein level changes. The protein content was extracted, and a Pierce BCA Protein Assay Kit (23327, Thermo Scientific, Rockford, USA) was used to determine the protein concentration. Equal amounts (20 µg) of protein for each group were resolved using an SDS-PAGE Gel Quick Preparation kit (Beyotime Biotechnology Co, China) and transferred onto PVDF membranes. The membranes were probed with primary antibodies in 0.01 M Tris-buffered saline supplemented with Tween-20 containing 5% bovine serum albumin, washed 3 × 8 min with TBST and followed by HRP-conjugated secondary antibody and TBST wash. When necessary, the PVDF membranes were stripped with Western blot stripping buffer (Thermo Scientific, Rockford, USA) for 30 min at room temperature, washed twice in TBST, and then re-probed. The protein bands were detected with Amersham ImageQuant 800 (GE Healthcare Life Sciences, Pittsburgh, USA). All specific protein band densities were normalized to GAPDH as the loading control and analyzed with ImageJ (1.45 s, National Institute of Health, US). Paired *t-test *was applied for statistical analysis, with the relative values of AOH eye calculated as divided by the values of the control eye.

### Intravitreal injection

The intravitreal injection was performed as previously reported [20] with micropipettes connected to an injector, and the speed of injection controlled by a pump. CTB-555 was injected bilaterally at a dose of 0.8 µL per eye 3–5 days before animal sacrifice. TRPA1 agonist polygodial (ab141518, Abcam, Cambridge) was given unilaterally at 100 µM for 1.5 µL, while the contralateral eye was given saline equivalent, and the retinae were collected 1 week post-injection. KN-93 (S6787, Selleck) was given unilaterally at 50 µM for 1.5 µL 3 days post AOH modeling, while the contralateral eye was given saline equivalent. HC-030031 (HC, ab120554, Abcam, Cambridge), a TRPA1 inhibitor could strongly reduce the mechanically evoked action potential firing in neural fibers in response to sustained force [23].To test the effect of HC-030031 on RGCs under AOH, animal models in this work were randomly divided into two groups: the control group received intravitreal injections of 1.5 µL sterile phosphate-buffered saline (PBS) bilaterally, while the treatment group received intravitreal injections of 1.5 µL 100 µM HC-030031 unilaterally on the right eye (model eye), and 1.5 µL PBS on the left eye (control eye) on post-AOH day 4.

### Calcium imaging

To test intracellular calcium concentration upon certain stimulation, healthy retina with PBS treatment were set as control for AOH or drug-treated groups, while those AOH retinae expressing TRPA1 were set as control for *Trpa1* knock-out ones. Eye balls were dissected, and retinae were mounted on 12-well plate containing bath solution prepared by mixing 88 mg of powdered Ames’ medium (Sigma) with 10 mL of H_2_O and 19 mg of sodium bicarbonate (23 mM). The medium was then loaded with either PBS or 10 µM HC-030031 for 30 min, then Furo-4AM (#S1060, 10 µM, Beyotime, China) for 30 min at 37 °C, and washed with the bath solution for 3 times. For calcium imaging of AOH retina, mice underwent unilateral AOH modeling for 80 min, after which both the control and AOH retinae were immediately collected and mounted for further procedures (i.e., day 0 of AOH modeling). The images were captured with a fluorescence microscope (BZ-X810 All-in-one Fluorescence Microscope, Olympus Corporation, Tokyo, Japan), with the time-gain-channel wavelength set at 1s-34-488 nm, at magnification x20 (NA = 0.45). Z-projected images were generated using BZ-X800 Analyzer (Version 1.1.2.4). One-way ANOVA with unpaired *t-test* was applied for statistical analysis.

### Immunofluorescence staining and enumeration of RGCs

The eye contralateral to AOH eye from the same individual was set as the control in the immunofluorescence tests. In *Trpa1* knock-out or HC antagonist tests, those unilateral AOH mice with RGCs expressing TRPA1 or intravitreously injected with PBS were set as control groups. Immunofluorescent labeling was performed on both retinal cryosections (5 μm) and retina flatmounts from 2-week AOH samples. Eyeballs with optic nerve were removed after transcardial perfusion of both PBS and 4% paraformaldehyde (PFA). To obtain frozen sections of the retina, eye cups were dissected and rinsed in PBS, immersed in sucrose solutions with increasing concentrations (10–30%), then embedded in OCT medium (4583, SAKURA Tissue-Tek, USA) and stored in 4 °C overnight. Eye cups were sectioned using frozen slicer apparatus (CM1950, Leica, Solms, Germany).

Cryosections were then stained following traditional immunostaining methods. Briefly, sections underwent antigen retrieval and blocking (20X sodium citrate diluted to 100 mL for retrieval followed by PBS containing 0.01% Triton X-100 (PBST), 5% BSA, and 5% donkey serum for blocking), and then primary antibody reaction with the appropriate secondary antibodies (Key resource table). The samples underwent 3 × 8 min PBS wash after each antibody immersion process. For immunofluorescence analysis of retinal flatmounts, the retinae were collected, washed in PBS, blocked in 0.01% PBST + 5% BSA + 5% donkey serum overnight at 4 °C, and then incubated with primary antibodies for 48 h and corresponding secondary antibodies (see Key resource table antibody details) overnight at 4 °C. After antibody incubation, retinae were washed three times in 0.1 M PBS, 30 min for each, before flat mounting with the RGC layer face up for further imaging. The central retina was identified as the region within 1.5 mm from the optic disc, and the peripheral retina was identified as the region around 3 mm from the optic disc (Fig. 1P). The RGC density of each retina was counted from 32 ROIs: four from the central region and four from the peripheral region retina for each quadrant. RGC densities (cells/mm^2^) were calculated by mean value of RGC counts of four ROIs from each quadrant in either the central or peripheral retina. Paired t-test was applied for statistical analysis, with the relative values of AOH eye calculated as divided by the values of the control eye.

### TUNEL assay

Terminal deoxynucleotidyl transferase dUTP nick end labeling (TUNEL) assay was applied for detection of RGC apoptosis on retinal slices 1 week after AOH modeling or polygodial administration. The eye contralateral to AOH or polygodial-injected eye from the same individual was set as the control. The DeadEnd Fluorometric TUNEL System G3250 kit was used to detect RGC apoptosis according to the manufacturer’s instructions (Promega, Madison, WI, USA). In brief, retinal cryosections were washed in PBS, intubated with PBS containing 0.1% Triton X-100 (PBST) for 15 min at 37 °C for membrane perforation, and again washed in PBS before enzymatic intubation for 1 h at 37 °C. The retinas were incubated with DAPI before being sealed for further immunofluorescence analysis.

### Image acquisition, processing and analysis

Images were obtained with a fluorescence microscope (BZ-X810 All-in-one Fluorescence Microscope, Olympus Corporation, Tokyo, Japan), equipped with four lasers (405 nm, 488 nm, 561 nm and 647 nm). Images of all retinal flatmounts or retinal slices were firstly acquired at low magnifications (x2 or x4, Plan Apochromat lenses) to help orientate and identify the regions of interest (ROIs). Image stacks (0.30–0.35 μm interval) were acquired for the ROIs using high magnification x10 (NA = 0.45), x20 (NA = 0.75) and x40 lenses (NA = 0.95). Z-projected images were generated using BZ-X800 Analyzer (Version 1.1.2.4).

Images for detecting TRPA1 expression in RGCs were acquired under the same laser exposure time and black balance index to enable comparison across sections. Specifically, the time-gain-channel wavelength was set at 1/20s-34-405 nm, 1/2s-115-488 nm and 1/6s-49–561 nm at magnification x40. The analysis of TRPA1 fluorescence intensity in RGC and the measurement of GCL + IPL + INL thickness were performed via Image J (1.45 s, National Institute of Health, US). For the TRPA1 fluorescence intensity measurement, threshold was set at 25 to 255 for 8-bit images. At least three background corrected mean gray values were pooled to average as the final mean fluorescence (mean ± SD). Line tool was used for the thickness measurement of the inner retina, namely GCL, IPL and INL for evaluation of RGC damage under AOH. For RGC counts, either in the central or peripheral retina, at least four regions of interest (ROI) in each quadrant were selected using polygon tool. RGC number in each ROI was manually counted to obtain average RGC counts in each quadrant, and these values were then pooled to average as their final mean RGC counts of each retina (mean ± SD).

Each intensity, thickness or number value was normalized by comparison between AOH eye and control eye in every individual, named self-control.

### Tissue optical clearing

The whole mouse brain was optically cleared by our previously reported protocol [20]. Briefly, mice 2 weeks after unilateral AOH were perfused intracardially with 20 mL 0.1 M PBS, followed by 4% PFA fixation. Brains were collected and immersed in 4% PFA for post-fixation at 4 °C for 1 day. Samples were then washed in PBS for 3 times, 2 h each on a shaker. Stepwise dehydration in a series of methanol in PBS (20%, 40%, 60%, 80%, 100%, one hour each at room temperature) and incubation with 66% DCM/33% methanol at 4 °C overnight were prepared. The final step was reflex index calibration, in which the samples were immersed in dibenzyl ether until clear (usually 1 to 2 days) to be prepared for the 3D imaging.

### Lightsheet 3D imaging and image analysis

Three-dimensional (3D) fluorescence imaging was performed using a lightsheet microscope (LS-18, Nuohai Co., Ltd) based on the protocol of Lu, et al. [20]. Briefly, a 6.3X/0.25 NA lens with bilateral illumination of 561 nm excitation wavelength. Images were captured using an ORCA Flash 4.0 camera with a resolution of 1 μm in the x and y axes and 2.5 μm in the z axis. The imaging was tiled six times to obtain the global focus. Imaris software (version 9.8, Oxford Instruments PLC, UK) were used for the 3D rendering and quantitative analysis. In this study, *Surface* algorithm was primarily used for fluorescence quantification. At least 3 samples were analysed in each group, with the relatively healthy side of the brain (to which the healthy RGC axons project) set as self-control, with the relative values of AOH side of the brain calculated as divided by the values of the control side. Paired *t-test* was applied for statistical analysis. In *Trpa1* knock-out or HC antagonist tests, however, those unilateral AOH mice with RGCs expressing TRPA1 or intravitreously injected with PBS were set as control groups, with the “relative values” of the AOH sides compared with the control groups. In that case, unpaired *t-test* would be applied for statistical analysis.

### Retrograde labelling of PrGPC- and PrGMC-projecting RGCs

Retrograde tracing of PrGPC- and PrGMC-projecting RGCs was reported previously [20]. In brief, mice were anesthetized with the fur of the skull shivered. They were then correctly positioned and fixed on the stereotaxic apparatus, ready for craniotomy. The skull was thinned over the target area (PrGPC: *x* = + 2.30 mm, *y*=-2.25 mm, *z*=-3.40 mm; PrGMC: *x* = + 2.55 mm, *y*=-2.25 mm, *z*=-3.40 mm) using a hand-held drill, and then equal volumes of CTB-555 (200 nl, BrainVTA) and CTB-488 (200 nl, BrainVTA) were unilaterally injected into the PrGPC and PrGMC respectively in C57BL/6J mice with or without AOH modelling. The micropipette was withdrawn slowly 2 min after injection, and the skin was sutured and given antibiotic ointment. Five days later, the animals were sacrificed for collecting the retina, optic nerve and brain.

### Quantification and statistical analysis

The specific quantification methods of corresponding items were mentioned above. All statistical analyses were performed with GraphPad Prism v8.0.2 (La Jolla, CA, USA) and means ± standard deviation (SD) were shown in the figures. The Shapiro–Wilk test was first used to verify the normal distribution of all the datasets, and the differences among groups were assessed with one-way analysis of variance (ANOVA) followed by Dunnett’s multiple comparison tests and two-tailed Student’s (*t-test*). * *P* < 0.05, ** *P* < 0.02 and *** *P* < 0.01 were considered significant.

## Results

### RGC degeneration under AOH

For establishment of AOH models, anterior chamber was cannulated (Fig. 1A). Corneal edema was observed upon the increase of IOP (Fig. 1B and Additional File 1: Fig. S1A-G). Four days after the IOP-elevation attack, the edematous cornea recovered with no more visible anterior segment anomaly (Fig. 1B). The retina was not that ischemic during the whole process of perfusion either, as non-perfused retinal vessels was not detected under a pre-set lens (Additional File 1: Fig. S1H-M). On day 7 post-AOH, mice underwent open field test (OFT) to assess the behavioral status, thereby indicating the traumatic effect of AOH, with AOH mice being more reluctant to move (total distance travel: 53.36 ± 8.62 m versus 35.75 ± 7.89 m; percentage of total immobile episodes: 18.56 ± 5.74% versus 30.82 ± 10.09%, Fig. 1C-F) and less willing to explore (number of zone cross: 60.38 ± 15.58 versus 31.00 ± 12.34, Fig. 1G-H), indicating anxious status due to impaired vision. AOH group also showed significantly prolonged P1 latency (Fig. 1L) and a decrease in P2-N1 amplitude (Fig. 1M) in F-VEP, suggesting impaired visual function (Fig. 1I-J and L-M). Retinae were then collected and prepared for both morphological and functional tests (Fig. 1B). Immunofluorescence staining showed retina thinning on retinal slices, especially the inner retina composed of ganglion cell layer (GCL), inner plexiform layer (IPL) and inner nucleus layer (INL) (AOH vs. control: 0.62 ± 0.04 vs. 1.00 ± 0.00 for relative thickness, *P* < 0.0001 (*n* = 6), Fig. 1N-O). RGC density was calculated at both the center and the periphery, showing reduced RGCs in retinal flatmounts at both the center (absolute counts 3753 ± 309 per mm^2^ (*n* = 4) for control and 2643 ± 193 per mm^2^ (*n* = 4) for AOH) and periphery (absolute counts 3817 ± 194 per mm^2^ (*n* = 4) for control and 2696 ± 328 per mm^2^ (*n* = 4) for AOH, Fig. 1P-R). Increased RGC degeneration 1 week after AOH was also indicated by cell apoptosis via TUNEL assay (Fig. 1S). All these results confirmed the damage to the RGCs under AOH.

### TRPA1 activation exacerbates RGC degeneration under AOH

To identify the potential IOP-sensitive molecules that participates in AOH-induced RGC degeneration, we performed RT-qPCR screening of the AOH-treated mice retinae comparing with the control (Fig. 2A). With the previous studies reporting the activation of several ion channels from PIEZO and TRP families under IOP elevation [24], we examined all the genes from PIEZO and TRP families searching for up-regulated genes, among which TRPA1 outstands in the AOH retinae (Fig. 2B and C).

To explore the time-course expression of TRPA1, RT-qPCR at different time points after AOH showed TRPA1 mRNA increased at about 7 days after AOH (Fig. 2D, 2.12 ± 0.60 for AOH retina compared with the control retina, *P* = 0.0006, *n* = 5), whereas the elevated protein level of TRPA1 was verified by western blot of 2-week retina samples (2.04 ± 0.70 for AOH retina compared with the control retina, *P* = 0.015, *n* = 6, Fig. 2E). Then, focusing on RGCs, immunofluorescent labeling of both the retinal slices and flatmounts indicated co-localization of TRPA1 on the GCL and with RGC membrane (Fig. 2F-G). We then calculated the relative fluorescence intensity of TRPA1 in GCL, revealing increased TRPA1 expression under AOH compared to the control eyes (Fig. 2H-I). Flow cytometry analysis of retinal cells indicated the increase of TRPA1 expression in RGCs under AOH (1.38 ± 0.32 for relative TRPA1 intensity of AOH group, *P* = 0.032, *n* = 6, Fig. 2J-N), together with the elevated percentage of TRPA1-positive RGCs (Fig. 2M). All these results pointed to the involvement of TRPA1 in RGC degeneration under AOH.

**Fig. 3:**
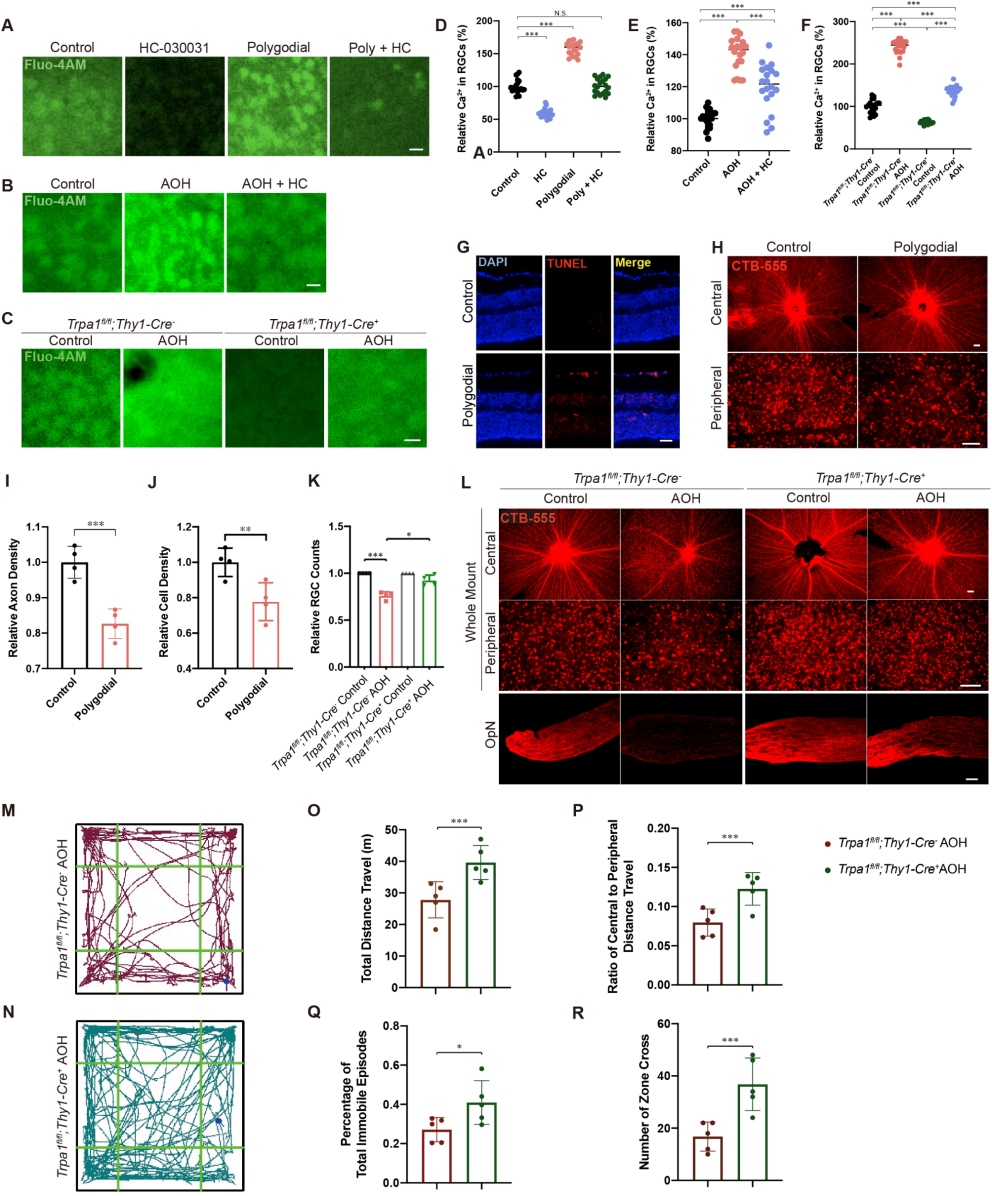
Traumatic effect of TRPA1 activation on RGCs. (**A**) Calcium imaging of fresh retinae revealed RGC responses in the control, HC (antagonist), Polygodial (TRPA1 agonist), and Polygodial + HC groups. Normalized Ca^2+^ signal of 20 randomly selected RGCs from three independent tests in different treatment groups were calculated in **(D)** (*n* = 20 for each group, unpaired *t-test*, ***P < 0.01). **(B)** Calcium imaging of fresh retinae showing RGCs from control group, AOH group and AOH + HC group right after modeling, with statistical analysis in **(E)** (*n* = 20 for each group, unpaired *t-test*, ***P < 0.01). **(C)** Calcium imaging with **(F)** statistical analysis in *Trpa1*^*fl/fl*^;*Thy1-Cre*^*−*^ control group, *Trpa1*^*fl/fl*^;*Thy1-Cre*^*−*^ AOH group, *Trpa1*^*fl/fl*^;*Thy1-Cre*^*+*^ control group and *Trpa1*^*fl/fl*^;*Thy1-Cre*^*+*^ AOH group (unpaired *t-test*, all *P* < 0.01). **(G)** TUNEL staining of retinal slices showing cell apoptosis among GCL and INL 1 week after intravitreal delivery of 100 µM polygodial or saline. Scale bar: 25 μm. **(H)** CTB-labeled RGCs and their axons at central and peripheral retina after intravitreal injection of polygodial or saline. CTB was administered 4 days before enucleation. Scale bar: 25 μm. **(I)** Relative RGC axon density in the control and polygodial-treated retina (*n* = 4 for each group, ***P < 0.01). **(J)** Relative RGC cell density in the control and polygodial-treated retina (*n* = 4 for each group, **P < 0.02). **(K)** Relative RGC counts in the control and AOH retina in *Trpa1*^*fl/fl*^;*Thy1-Cre*^*−*^ and *Trpa1*^*fl/fl*^;*Thy1-Cre*^*+*^ mice (*n* = 4 for each group, *P < 0.05, ***P < 0.01). **(L)** CTB-labelled RGC soma and axons shown on retinal flatmount and optic nerve slices. CTB was administered 4 days before enucleation. **(M-N)** Track plots of **(M)***Trpa1*^*fl/fl*^;*Thy1-Cre*^*−*^ and **(N)***Trpa1*^*fl/fl*^;*Thy1-Cre*^*+*^ AOH mice in the open field. **(O-R)** Comparison of **(O)** total distance travelled, **(P)** percentage of total immobile episodes, **(Q)** ratio of central to peripheral distance travelled and **(R)** number of zone cross between *Trpa1*^*fl/fl*^;*Thy1-Cre*^*−*^ and *Trpa1*^*fl/fl*^;*Thy1-Cre*^*+*^ AOH mice (*n* = 5 for each group, unpaired *t-test*, *P < 0.05, ***P < 0.01). Results are presented as mean ± standard deviation (SD). AOH, acute ocular hypertension

Then, to further explore the causal relationship between TRPA1 activation and RGC degeneration, functional activation and inhibition of TRPA1 was conducted. First, we found increased Ca^2+^ influx in RGCs immediately after AOH, which was significantly suppressed by TRPA1 selective antagonist HC-030031 (Fig. 3B and E). The RGC calcium influx could also be stimulated by TRPA1 agonist Polygodial, simulating the [Ca^2+^] elevation under AOH, yet being inhibited to control level under combination of Polygodial and HC (Fig. 3A and D). The cellular specificity of Fluo-4AM labeling was confirmed by post-imaging fixation and co-immunostaining (Additional File1: Fig. S3). The long-term traumatic effect of TRPA1 activation was verified by both TUNEL staining (Fig. 3G), and CTB-labeled RGCs and their axons at central and peripheral retina 1 week after intravitreal injection of 100 µM polygodial (Fig. 3H), showing RGC apoptosis as well as decrease in RGC somatic and axonal density (Fig. 3I-J).

Next, we conditionally knocked out *Trpa1* in RGCs by crossbreeding *Trpa1*^*fl/fl*^ mice with *Thy1-Cre*^*+*^ mice to produce *Trpa1*^*fl/fl*^;*Thy1-Cre*^*+*^ genotype (Additional File1: Fig. S2A). The successful *Trpa1* cKO in nearly all RGCs was verified by immunostaining (Additional File1: Fig. S2B), together with PCR results of constitutive KO allele showing tissue-specific gene deletion (Additional File1: Fig. S2C-D). Functionally, calcium imaging indicated partly inhibited calcium influx in RGCs immediately after AOH when conditionally knocked out *Trpa1* in RGCs (Fig. 3C and F). We then collected the retina, optic nerves and brains two weeks after AOH for either immunofluorescent staining and CTB labeling. The results showed a decrease of the RGC axonal density in the central retina, and a decrease of RGC cell count in the peripheral retina in *Trpa1*^*fl/fl*^;*Thy1-Cre*^*−*^ mice (Fig. 3L) under AOH, and this decrease was partially reversed when *Trpa1* was conditionally knocked out in RGCs (Fig. 3K and L; relative RGC counts compared with control eyes: 0.76 ± 0.04 versus 0.92 ± 0.06 for *Trpa1*^*fl/fl*^;*Thy1-Cre*^*−*^ and *Trpa1*^*fl/fl*^;*Thy1-Cre*^*+*^ mice, respectively. *P* = 0.038). Finally, alleviated anxiety status related to visual impairment were observed in *Trpa1*^*fl/fl*^;*Thy1-Cre*^*+*^ AOH mice (Fig. 3M-R).

**Fig. 4:**
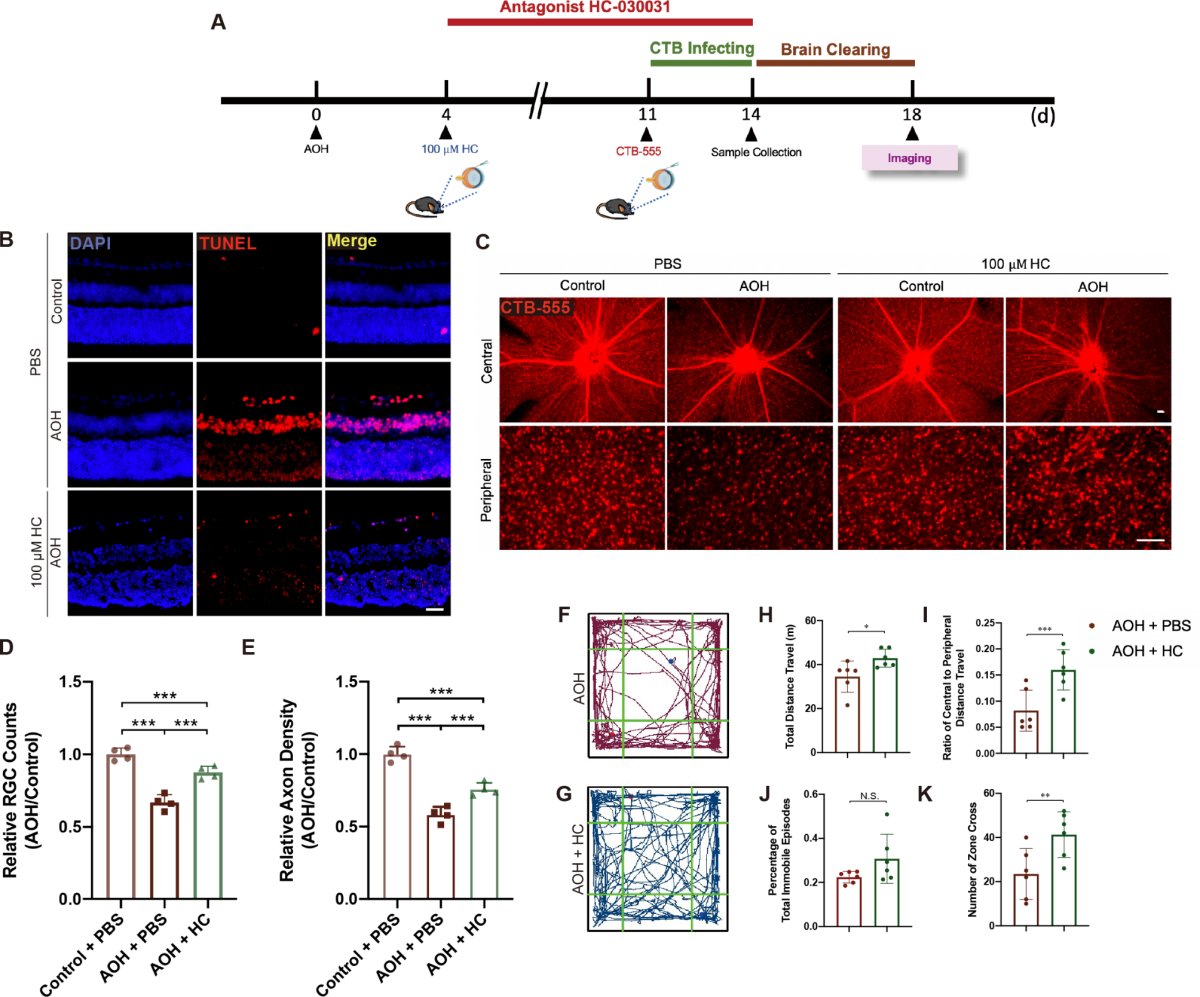
HC showed protective effect on RGC. (**A**) Experimental design. **(B)** TUNEL staining of retinal slices showing decreased apoptosis after intravitreal delivery of 100 $\:{\upmu\:}$M HC. Scale bar: 25 μm. **(C)** Retinal flatmounts showing RGCs and their axons under control, AOH, and AOH + HC conditions. Scale bar: 50 μm. **(D)** Relative RGC counts and **(E)** RGC axon density calculated in the CTB-labeled retina in the center and periphery (*n* = 4 for each group, paired *t-test*, **P < 0.02, ***P < 0.01). **(F)** Track plots of AOH mice treated with PBS or **(G)** HC in the open field. **(H-K)** Comparison of **(H)** total distance travelled, **(I)** ratio of central to peripheral distance travelled, **(J)** percentage of total immobile episodes, and **(K)** number of zone cross between PBS and HC-treated AOH mice (*n* = 6 for each group, unpaired *t-test*, *P < 0.05, **P < 0.02, ***P < 0.01). Results are presented as mean ± standard deviation (SD). AOH, acute ocular hypertension

Finally, to explore the potential protective effect of blocking TRPA1, 100 µM HC-030031 (HC) was given intravitreally 4 days post-AOH (Fig. 4A). Then, in the treatment group where HC was given in the AOH eye, alleviated cell apoptosis (Fig. 4B), and slightly increased central axonal density and peripheral RGC counts in the retina (Fig. 4C) were detected. The treatment group, however, did not reach complete recovery, indicating other potential mechanisms involved in AOH pathogenesis (Fig. 4D and E, relative axon density of 0.58 ± 0.05 for AOH eyes treated with PBS, 0.76 ± 0.05 for AOH eyes treated with equivalent 100 µM HC, *P* = 0.003; relative cell counts of 0.67 ± 0.05 for AOH eyes treated with PBS, 0.88 ± 0.04 for AOH eyes treated with 100 µM HC, *P* = 0.001). HC also partly rescued vision loss-related anxious behaviors after AOH (Fig. 4F and G), and significantly protected visual function in terms of the P1 latency and P2-N1 amplitude in F-VEP (Fig. 1K and M). All these results confirmed the traumatic effect of TRPA1 on RGCs under AOH.

### TRPA1 exacerbates RGC degeneration under AOH through the Ca(2+)/CaMKII/CREB pathway

**Fig. 5:**
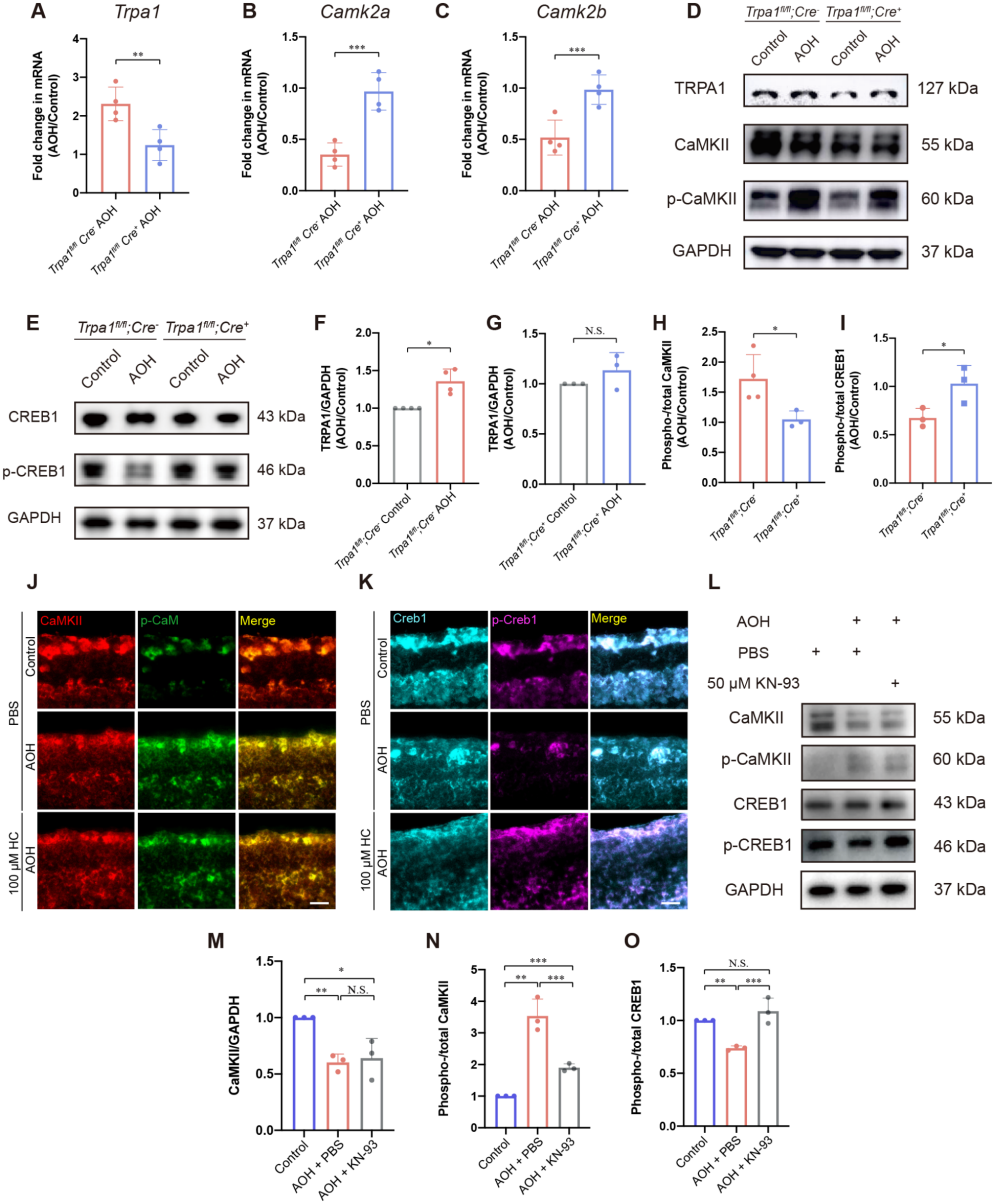
Effect of TRPA1 activation on the retinal expression of Ca(2+)/CaMKII/CREB signaling pathways under AOH. (**A**-**C**) The mRNA level of (**A**) *Trpa1*, (**B**) *Camk2a* and (**C**) *Camk2b* in retina at day 7 in AOH eyes without or with conditional knock out of *Trpa1* in RGCs compared with the contralateral control eyes (*n* = 4 for each group, unpaired *t-test*, **P < 0.02, ***P < 0.01). **(D)** Western blot and the relative optical densities analysis of the level of TRPA1 in **(F)***Trpa1*^*fl/fl*^;*Thy1-Cre*^*−*^ group and **(G)***Trpa1*^*fl/fl*^;*Thy1-Cre*^*+*^ group without or with AOH (*n* = 4 for *Trpa1*^*fl/fl*^;*Thy1-Cre*^*−*^ group, *n* = 3 for *Trpa1*^*fl/fl*^;*Thy1-Cre*^*+*^ group, paired *t-test*, *P < 0.05). **(H)** The relative phosphorylation of CaMKII (calculated as p-CaMKII/total CaMKII) in *Trpa1*^*fl/fl*^;*Thy1-Cre*^*−*^ group and *Trpa1*^*fl/fl*^;*Thy1-Cre*^*+*^ group (*n* = 4 for *Trpa1*^*fl/fl*^;*Thy1-Cre*^*−*^ group, *n* = 3 for *Trpa1*^*fl/fl*^;*Thy1-Cre*^*+*^ group, unpaired *t-test*, *P < 0.05). **(E)** Western blot and **(I)** the relative level of CREB1 phosphorylation (*n* = 4 for *Trpa1*^*fl/fl*^;*Thy1-Cre*^*−*^ group, *n* = 3 for *Trpa1*^*fl/fl*^;*Thy1-Cre*^*+*^ group, unpaired *t-test*, *P < 0.05). **(J)** Immunofluorescent staining of the inner retina (GCL + IPL + INL), showing co-localization and expression of CaMKII and p-CaMKII, and **(K)** CREB1 and p-CREB1. **(L)** Western blot and the optical densities analysis of the relative level of **(M)** CaMKII, **(N)** CaMKII phosphorylation and **(O)** CREB1 phosphorylation (*n* = 3 for each group, unpaired *t-test*, **P < 0.02, ***P < 0.01). GAPDH was used as the internal controls. All data were presented as mean ± SD. AOH, acute ocular hypertension

The threat TRPA1 activation posed on RGCs upon acute elevation of IOP resulted in RGC degeneration, yet the pathways downstream of TRPA1 remain to be elucidated. To identify the involvement of calcium-induced downstream pathways in the pathogenesis of TRPA1-mediated RGC degeneration, eyes of from *Trpa1*^*fl/fl*^;*Thy1-Cre*^*−*^ and *Trpa1*^*fl/fl*^;*Thy1-Cre*^*+*^ mice were applied for detection of key molecules at the retina level. RT-qPCR results showed that AOH significantly downregulated the *Camk2a* and *Camk2b* transcriptional level, and this effect was abolished when conditionally knocked out *Trpa1* in RGCs (Fig. 5A-C). Consistently, we observed the decrease of overall CaMKII level under AOH in *Trpa1*^*fl/fl*^;*Thy1-Cre*^*−*^ mice retina, yet this levelled up in *Trpa1*^*fl/fl*^;*Thy1-Cre*^*+*^ group. The elevated relative level of CaMKII phosphorylation indicated the activation of Ca(2+)/CaMKII signaling pathway (Fig. 5D, F-H), which served various biological process. Further, the downregulation of CREB1 phosphorylation under AOH as well as its recovery after *Trpa1* cKO pointed to the involvement of CREB pathway in AOH-induced RGC degeneration, downstream of TRPA1 (Fig. 5E and I). For identification of RGC-specific CaMKII or CREB changes, immunofluorescent observation of the inner retina (GCL + IPL + INL) confirmed the activation of Ca(2+)/CaMKII signaling pathway and the inhibition of CREB pathway in RGCs under AOH (Fig. 5J and K). The CREB1 inactivation downstream of CaMKII activation was further verified by intravitreal delivery of KN-93, an inhibitor of CaMKII phosphorylation, showing upregulated p-CREB1 level under AOH (Fig. 5L-O). Thus, the exacerbation of RGC degeneration under AOH through the Ca(2+)/CaMKII/CREB pathway was confirmed.

### TRPA1-mediated region-selective RGC central projection damage under AOH

**Fig. 6:**
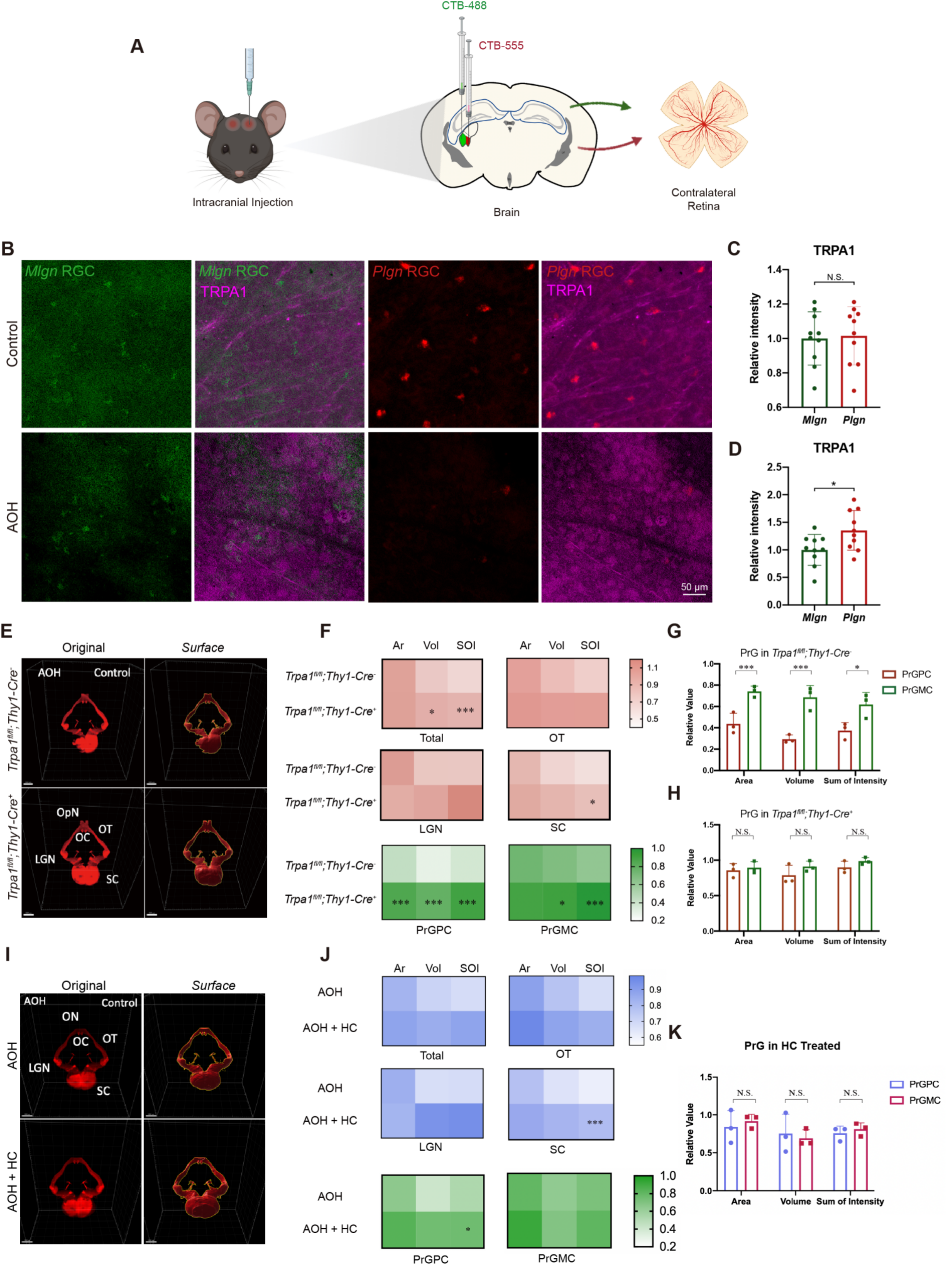
2D and 3D observation of TRPA-mediated selective RGC damage pattern. (**A**) Schematic illustration of brain stereotaxic injection of CTB-488 and CTB-555 for grouping of RGCs. **(B)** Immunostaining of retina flatmount showing distribution of *Plgn* RGC and *Mlgn* RGC together with TRPA1 expression under control or AOH conditions. Scale bar: 50 μm. *Plgn* RGC and *Mlgn* RGC were retrogradely labeled by CTB. Note that in all other conditions where RGCs and their central projections were labeled with CTB, an intravitreal dose of CTB was applied for anterograde labeling. **(C-D)** Comparison of the relative TRPA1 intensity between *Plgn* RGC and *Mlgn* RGC in **(C)** control retina and **(D)** AOH retina (*n* = 10 for each group, *P < 0.05, N.S., non-significant). **(E)** 3D-reconstructed whole brain showing the changes of RGC central projection under AOH with or without conditional knock-out of *Trpa1* in RGCs. Different brain regions were labeled, and the original data (left column) were surfaced (right column). AOH refers to the side of the brain where the RGCs of AOH model eye projected to. Scale bar: 1000 μm. **(F)** Heatmaps showing the relative value of the surface area, volume and sum of intensity of whole RGC central projection, OT, LGN, SC, and PrGPC, PrGMC in the AOH side compared with the control side in mice with or without conditional knock-out of *Trpa1* in RGCs (*n* = 3 for each group, *P* values stand for differences between *Trpa1*^*fl/fl*^;*Thy1-Cre*^*−*^ and *Trpa1*^*fl/fl*^;*Thy1-Cre*^*+*^ AOH side. *P < 0.05, **P < 0.02, ***P < 0.01). **(G-H)** Comparison of the extent of decrease in both the area, volume and sum of intensity between PrGPC and PrGMC (*n* = 3 for each experiment, paired *t-test*, **P < 0.02) in **(G)***Trpa1*^*fl/fl*^;*Thy1-Cre*^*−*^ and **(H)***Trpa1*^*fl/fl*^;*Thy1-Cre*^*+*^ mice. **(I)** 3D-reconstructed whole brain. AOH refers to the side of the brain where the RGCs of AOH model eye projected to. Scale bar: 1000 μm. **(J)** Heatmaps showing the relative value of the surface area, volume and sum of intensity of different brain regions in the AOH side of brains compared with the control side with or without HC intravitreal injection (*n* = 3, *P < 0.05, ***P < 0.01 for comparison of AOH and AOH + HC group). Comparison of the extent of decrease in both the area, volume and sum of intensity between PrGPC and PrGMC (*n* = 3 for each experiment, paired *t-test*, *P < 0.05) in AOH mice without or with HC treatment were also shown (*n* = 3, *P < 0.05). **(K)** Direct comparison of PrGPC and PrGMC under AOH after HC treatment (*n* = 3 for each experiment, unpaired *t-test*, n.s., non-significant). Ar, surface area; CTB, cholera toxin subunit B; DCM, dichloromethane; LGN, lateral geniculate nucleus; OC, optic chiasm; ON, optic nerve; OT, optic tract; PrGMC, magnocellualr part; PrGPC, parvocellular part; SC, superior colliculi; SOI, sum of intensity; Vol, volume

Neuronal selective vulnerability was previously evaluated by functional and morphological tests, yet the further exploration of the mechanisms behind called for more precise approaches [25–27]. Based on our previously reported protocol, we were able to group RGCs in terms of their varied axonal vulnerability after AOH attack [20]. Further, we questioned the relationship between TRPA1 and RGC selective damage under AOH. As PrGPC and PrGMC showed distinct vulnerability by 3D observation, these two brain regions where RGC axons project to were exemplified for exploring the mechanisms involving TRPA1. *Plgn* RGC and *Mlgn* RGC were retrogradely labeled by CTB-555 and CTB-488 (Fig. 6A) with immunofluorescent co-localization test showing semi-quantitatively the more profound upregulation of TRPA1 in *Plgn* RGC under AOH (Fig. 6B-D). We further observed changes in the RGC central projection to test if the damages were alleviated or the region-selective pattern disappeared in *Trpa1* cKO mice. The results indicated that cKO of *Trpa1* could partly reverse the loss of RGC axon degeneration in their projection to OT, LGN, SC, and the total RGC central projection (Fig. 6E-F; Additional File1: Fig. S4A-D, Table S3). We also found that although selective tendency of damage on PrGPC and PrGMC could still be observed under AOH, they were, however, rescued to a comparable level in *Trpa1* cKO mice (Fig. 6F-G, PrGPC vs. PrGMC in *Trpa1*^*fl/fl*^;*Thy1-Cre*^*−*^ mice: 0.44 ± 0.10 vs. 0.74 ± 0.05 for the relative surface area, *P* < 0.01; 0.29 ± 0.04 vs. 0.69 ± 0.11 for the relative volume, *P* < 0.01 and 0.37 ± 0.08 vs. 0.62 ± 0.12 for the relative sum of intensity, *P* = 0.037. PrGPC vs. PrGMC in *Trpa1*^*fl/fl*^;*Thy1-Cre*^*+*^ mice: 0.86 ± 0.10 vs. 0.89 ± 0.09 for the relative surface area; 0.79 ± 0.14 vs. 0.91 ± 0.08 for the relative volume and 0.90 ± 0.09 vs. 0.99 ± 0.05 for the relative sum of intensity, all *P* > 0.05). The use of TRPA1 antagonist, HC, significantly protected SC in terms of the sum of intensity (Fig. 6I-J; Additional File1: Fig. S4E-H). The results of OT, LGN and total RGC central projection were, however, insignificant. Nevertheless, when again dividing the LGN into subregions, the partial recovery of both PrGPC and PrGMC, together with the elimination of region disparity between PrGs confirmed the therapeutic action of HC (Fig. 6J-K), as well as the involvement of TRPA1 in region-selective vulnerability of RGC axons. Altogether, these results confirmed that TRPA1 activation exacerbated AOH-induced region-selective RGC central projection damage, thereby reflecting the selective vulnerability of different RGC groups.

## Discussion

In this study, we for the first time confirmed the traumatic effect of TRPA1 activation and upregulation in the pathogenesis of AOH-induced vision loss, as well as the mechanisms behind. Our study also adds to the previous findings of neuronal selective vulnerability by demonstrating the role of TRPA1 in the region-specific damage pattern of RGC axons under AOH.

In accordance with the previous studies reporting upregulation of TRPA1 in retina ischemia-reperfusion injury [19], we confirmed the increased expression of TRPA1 in AOH retina among other TRP and PIEZO family members, accompanied with its activation, thereby inducing RGC degeneration. The upregulation of TRPA1 could be due to the activation of certain transcription factors, thereby inducing the intra-nucleus transcription of TRPA1 mRNA, as well as the final TRPA1 density on cell membrane. As a non-selective cation channel with a much higher permeability to calcium compared to other groups of TRPs [12, 17], TRPA1 is responsible for the depolarization of the membrane when activated [28]. This depolarization on the one hand increases cell excitability, and on the other hand, the massive influx of Ca^2+^ may result in cell excitotoxicity via various Ca^2+^-triggered downstream pathways [29]. This Ca^2+^ overload exacerbates mitochondrial dysfunction, reactive oxygen species (ROS) production, and endoplasmic reticulum (ER) stress in RGCs, accelerating apoptotic pathways [30]. TRPA1 activation also amplifies neuroinflammation by releasing pro-inflammatory mediators (e.g., substance P) and sensitizing NMDA receptors, further promoting excitotoxic damage [31]. Additionally, TRPA1-mediated signaling disrupts axonal transport and synaptic connectivity, contributing to RGC degeneration [32].

TRPA1 activation induces substantial Ca^2+^ influx, triggering downstream pathways, including the Ca^2+^/CaMKII/CREB axis investigated here. Though as a central coordinator of Ca^2+^ signal transduction [33], it remains conflict how CaMKII regulate RGC functions under physical and pathological conditions. Normally CaMKII is a multifunctional serine-threonine protein kinase that involves in a variety of neuronal functions [34]. In our study, through quantitative analysis of mRNA and protein level changes under AOH, we found the overall CaMKII level decreased, in accordance with recent findings regarding loss of CaMKII upon axonal degeration [35, 36]. However, we also detected an increase in the relative phosphorylation of CaMKII (p/t-CaMKII). The upregulated phospho-CaMKII might exacerbate RGC degeneration by promoting pro-apoptotic signaling pathways, mitochondrial dysfunction [37], and oxidative stress [37]. Phosphorylated CaMKII amplifies excitotoxic damage by enhancing NMDA receptor activity and destabilizing calcium homeostasis [38], further driving caspase-3 activation and apoptotic cell death. Additionally, CaMKII-mediated phosphorylation disrupts synaptic integrity and axonal transport in RGCs, accelerating neurodegeneration [39]. Looking back at our findings, the downregulated CREB1 phosphorylation should be interpreted with care. Under acute ocular hypertension, hyperactivation of CaMKII via phosphorylation could exert dual effects on CREB1, a transcription factor critical for neuronal survival. Normally, CaMKII phosphorylates CREB1 at Ser133, promoting its binding to CREB-binding protein (CBP) to activate pro-survival genes (e.g., Bcl-2, BDNF). However, in pathological calcium overload caused by acute intraocular pressure spikes, on the one hand, excessive CaMKII signaling exacerbates mitochondrial dysfunction and oxidative stress [34, 37]. This may affect though inhibition of adenylate cyclase (AC)-cAMP-protein kinese A (PKA) pathway, which impair CREB1 phosphorylation and nuclear translocation, diminishing its transcriptional activity. Concurrently, CaMKII-driven excitotoxicity via NMDA receptor overactivation could shift CREB1 signaling toward pro-apoptotic pathways, upregulating stress-responsive genes (e.g., Bax, FasL) that promote RGC death [40]. This imbalance-loss of CREB1-mediated survival signals alongside amplified apoptotic cues-accelerates RGC degeneration. And since both genetically knock out of *Trpa1*, and intravitreous administration of TRPA1 antagonist HC-030031 help restored the phophorylation of both CaMKII and CREB1, the activity of Ca(2+)/CaMKII/CREB pathway downstream of TRPA1 was confirmed. The neuroprotective effect of TRPA1 antagonist, HC, on the other hand, suggest TRPA1 as a potential therapeutic target in clinical practice.

Apart from general degeneration of neurons, the selective vulnerability of neurons has been drawing far more attention with regard to its clinical significance in providing individualized therapies for target patients. The tendency of neurons being more vulnerable to trauma could be due to either structurally the size and shape of the soma, or functionally the expression and distribution of certain molecules, either baseline or trauma-induced. In this study, the role of TRPA1 on the clinically and functionally-found selective RGC vulnerability under ocular hypertension was further questioned and explored. The single-cell transcriptomics enabled new breakthroughs in the field of studying neural selective vulnerability [41], but we here put forward a new methodology to magnify the varied degree of RGC degeneration that was otherwise difficult to identify at the retina level [20]. Our modified whole-brain clearing method, optimized for speed and fluorescence preservation, enables comprehensive 3D mapping of RGC axonal arborizations across the entire brain. This approach overcomes spatial fragmentation inherent to section-based methods and allows precise quantification of region-specific axonal damage under AOH. By resolving subtle differences in degeneration between brain regions, we could directly link TRPA1 activation to selective vulnerability of RGC subtypes based on their central projection targets. By taking PrG in the LGN as an example, we succeeded in grouping RGCs according to their axonal damage, and thereby magnifying the varied degree of RGC degeneration in the retina level which were otherwise hard to identify. This paved the way for further investigations into the molecular mechanisms behind RGC selective damage. The immunofluorescent co-localization test confirmed the more profound upregulation of TRPA1 in *Plgn* RGCs, which partly accounted for them being more vulnerable, as indicated by our findings of traumatic TRPA1 activation and upregulation in RGCs. However, as RGC axonal central arbor in rodents differs from primates and that no precise corresponding M cells or P cells were discovered in rodents, the results may not able to directly reflect factors that account for neuronal selective vulnerability in primates. But since it was reported that the selective vulnerability still exists in different subtypes of $\:{\upalpha\:}$RGCs in rodents [42, 43], and that we noted the distinct cellular size between *Plgn* RGCs and *Mlgn* RGCs, there might be a potential similar retina-brain correspondence between rodents and primates.

In our work, with the help of rodents that were capable of gene regulation, by specifically knocking out *Trpa1* in RGCs, or intravitreously giving TRPA1-specific antagonist, we were able to see that the AOH-induced RGC axonal projection damage was much more alleviated (Fig. 6E), and that TRPA1 “contributes to” approximately 20% of RGC axonal degeneration under AOH (Additional File1: Table S3). The traumatic effect of TRPA1 under AOH was verified, and this in the meanwhile confirmed the exacerbation of TRPA1 in RGC selective vulnerability under AOH, and thereby reminding the therapeutic opportunity for both the neuroprotection and the elimination of pathway-selective neuronal damage under different modes of IOP elevation by targeting TRPA1 [35, 44]. Here, as confirmed in our work, the application of TRPA1 antagonists (e.g., HC-030031) could block Ca^2+^ entry, restoring calcium homeostasis and reducing downstream apoptotic pathways, and thereby preserving structural and functional integrity of RGCs. It could also restore CREB1 activity, rebalancing apoptotic and survival signals in RGCs. Given the potential neuroprotective effects of TRPA1 inhibition-such as preserving mitochondrial function, reducing reactive oxygen species (ROS) production [45], and mitigating neuroinflammation [46]-the findings of this study suggest promising clinical implications for targeting TRPA1. These include combining TRPA1 inhibitors with intraocular pressure (IOP)-lowering agents or neurotrophic factors (e.g., BDNF) to achieve multi-modal neuroprotection. Additionally, TRPA1 could serve as a biomarker to identify activation signatures (e.g., oxidative stress markers) or specific patterns of IOP elevation (e.g., acute, chronic, or normal-tension glaucoma), enabling the stratification of patients who are most likely to benefit from TRPA1-targeted therapies.

There are several limitations of this study, which in the meanwhile implicate directions for future research. Firstly, diverse models of different IOP elevation modes should be applied to testify the selective RGC changes, for example, the RGC central projection damage patterns under chronic ocular hypertension, or even normal tension glaucoma models. Secondly, despite the findings of TRPA1’s exacerbation on selective *Plgn* and *Mlgn* RGC vulnerability under AOH, other potential mechanisms as well as RGC functional abnormalities were far beyond discovery. Finally, the limited exploration into the ideal HC treatment concentration also left questions on whether a lower concentration of HC with less drug toxicity could be effective to protect RGCs from degeneration. The use of anesthetics should also be re-considered, as pharmaceutical-grade compounds, such as ketamine/xylazine, should be adopted for anesthesia in any future experiments involving the AOH model. Given the more comprehensive researches being conducted, the understanding of vision and vision loss under pathological conditions could be brought to a greater extent.

**Fig. 7:**
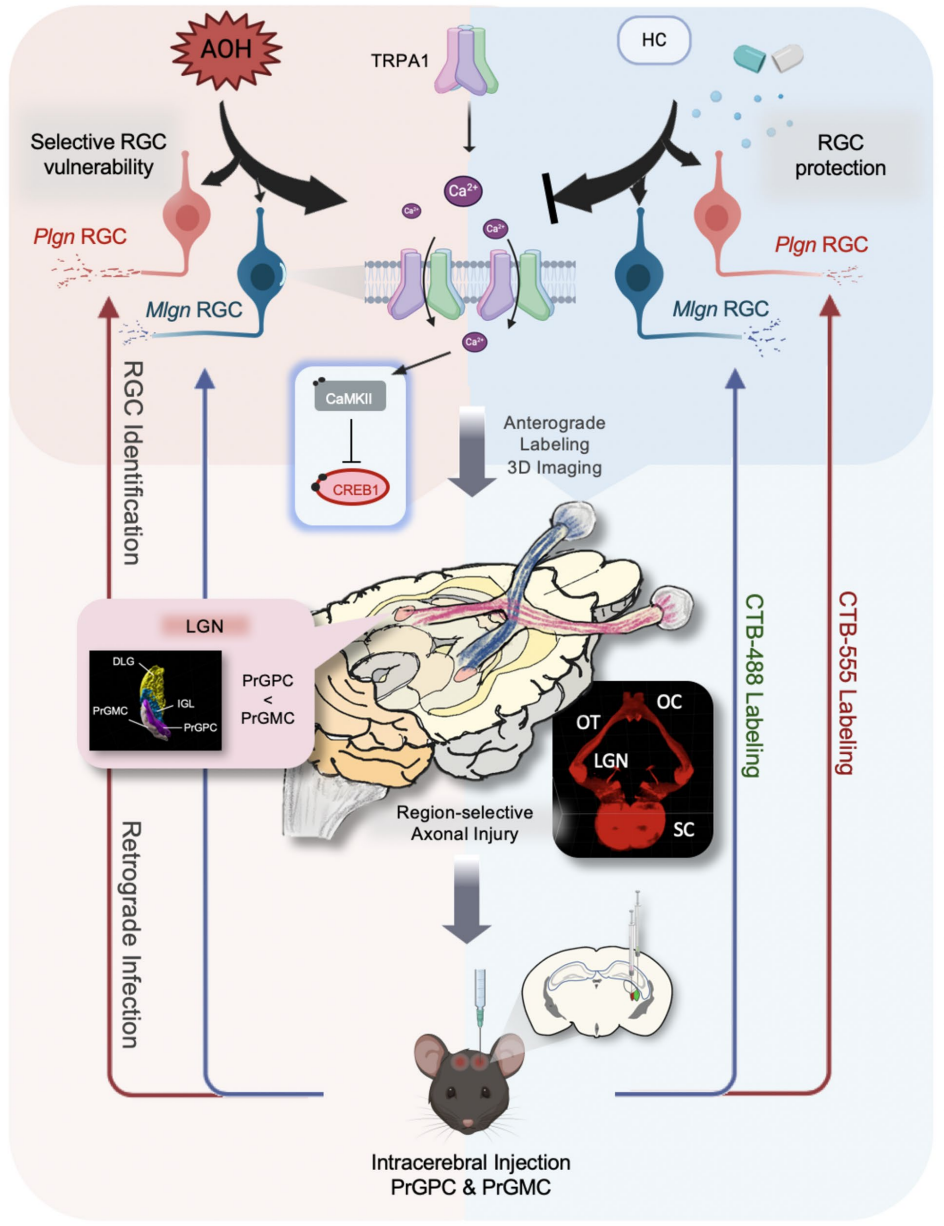
Graphical abstract

In summary, the present study suggests that TRPA1 plays a key role in RGC degeneration and by a novel tissue optical clearing approach, we confirmed its role in selective RGC vulnerability under AOH. Furthermore, our study provides insights into the pharmacological application of targeting TRPA1 in individualized treatment of neuroprotection in acute IOP elevation-induced RGC degeneration (Fig. 7).

## Electronic supplementary material

Below is the link to the electronic supplementary material.

Supplementary Material 1

Supplementary Material 2

## Data Availability

The datasets used and analysed during the current study available from the corresponding author on reasonable request.
