# Supplementary material for: TRPA1 exacerbates selective retinal ganglion cell vulnerability under acute ocular hypertension
Source: Acta Neuropathol Commun. 2025 Apr 5;13:70. doi: 10.1186/s40478-025-01974-5 (PMC11971892; doi:10.1186/s40478-025-01974-5)
Supplement: Supplementary file 1 — Supplementary Material 1 [file 40478_2025_1974_MOESM1_ESM.pdf]

Supplementary File 1 for

TRPA1 exacerbates selective RGC vulnerability under acute ocular hypertension

Wenhan Lu, Yu Wang, Wei Hu, Xinyi Lin, Xiaoyu Tong, Yi Tian, Yuning Chen, Yicong Wang, Yan Xiao, Hongfang Yang*, Yi Feng*, Xinghuai Sun*

Correspondence to: xhsun@shmu.edu.cn (Xinghuai Sun)

This PDF file includes:

Supplementary Text
Figs. S1 to S4
Tables S1 to S3

Supplementary Text

Confirmation of RGC calcium activity in calcium imaging

To confirm that the retinal calcium signals shown in Figure S3 referred to RGCs, retinal flatmount immunofluorescence staining was conducted right after calcium imaging following retinal fixation, blocking, primary antibody (Tuj1 for RGC labeling and Iba-1 for microglia labeling) and secondary antibody intubation. The results were showing the co-localization of Tuj1 signals with Fluo-4AM signals, yet Iba-1 showed no co-localization with Fluo-4AM fluorescence (Figure S3).

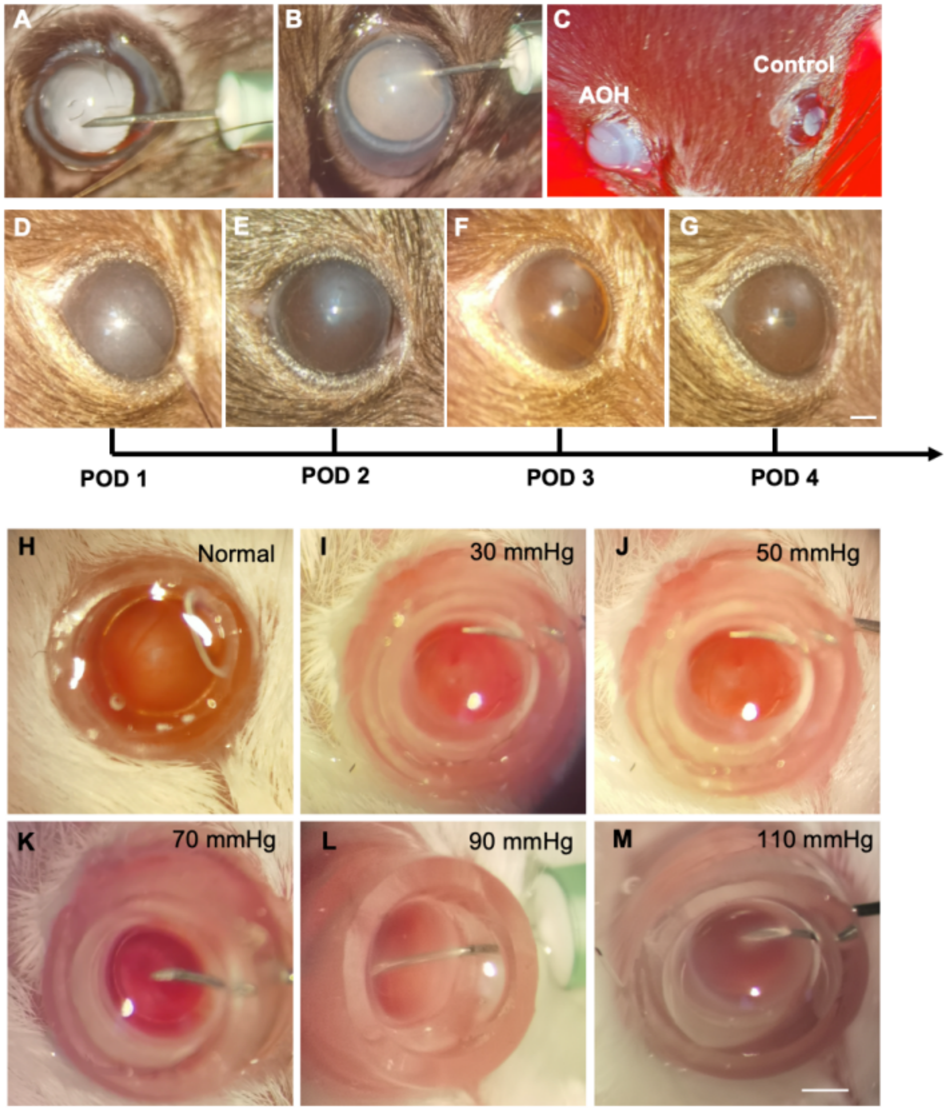

Figure S1. Acute ocular hypertension. (A) At the very beginning of anterior chamber cannulation with a set height of 97 cm for the saline reservoir, providing an approximately 70 mmHg IOP. Pupil was dilated with a mixture of 0.5% tropicamide and 0.5% phenylephrine eye drops. **(B)** 80 min after acute ocular hypertension. Cornea is apparently edematous. **(C)** Comparison of right AOH eye and left control eye. The contralateral left eye was cannulated without saline reservoir. **(D)** ~ **(E)** Picture of the anterior segment after AOH was taken at POD 1-4, showing recovery of corneal edema at approximately 4 days after AOH. **(F and G)** Test of the impact of elevated IOP on retinal blood perfusion using a balb/c mouse. The anterior chamber was cannulated, and the IOP was set accordingly while the fundus was imaged under a pre-set lens. The fundus was slightly blurry due to edematous cornea in **(K)**, and appeared whitish-grey in color due to non-perfusion in **(L)** and **(M)**. Scale bar indicates 1 mm. AOH, acute ocular hypertension; POD, post-operation day.

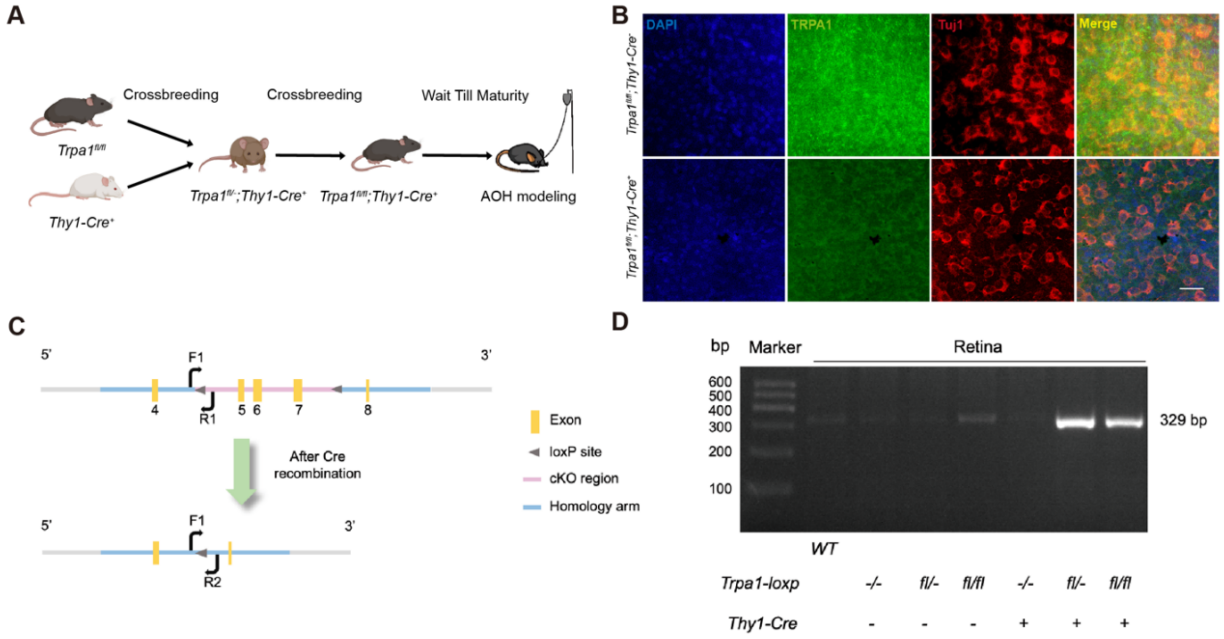

Figure S2. Genotype identification and efficacy confirmation of tissue-specific knock-out of *Trpa1*. (A) Schematic illustration of crossbreed strategy. *Trpa1*$^{fl/fl}$ mice were cossbred with *Thy1-Cre* mice to produce *Trpa1*$^{fl/fl}$*;Thy1-Cre*$^{+}$ genotype. **(B)** Immunofluorescent staining of retina flatmount from *Trpa1*$^{fl/fl}$*;Thy1-Cre*$^{-}$ or *Trpa1*$^{fl/fl}$*;Thy1-Cre*$^{+}$ genotype. Scale bar indicates 25 µm. **(C)** Schematic diagram showing *Trpa1* exons on chromosome, the loxP sites and the corresponding conditional knock out region. F1 and R1 refers to forward and reverse primers for genotype identification of *Trpa1*$^{fl/fl}$ mice. Numbers below the exons indicated the sequence of *Trpa1* exons on chromosome 1. After crossbreeding *Trpa1*$^{fl/fl}$ mice with *Thy1-Cre* mice to produce *Trpa1*$^{fl/fl}$*;Thy1-Cre*$^{+}$ genotype, RGC-specific knock out of *Trpa1* took place. F1 and R2, as indicated in the diagram, were applied for confirmation of gene recombination. **(D)** PCR results of retina samples collected from mice of different genotypes. F1 and R2 were used as primers for gene amplification, with the result showing the tissue-specific recombination occurring in *Trpa1*$^{fl/-}$*;Thy1-Cre*$^{+}$ and *Trpa1*$^{fl/fl}$*;Thy1-Cre*$^{+}$ genotype. cKO, conditional knock-out.

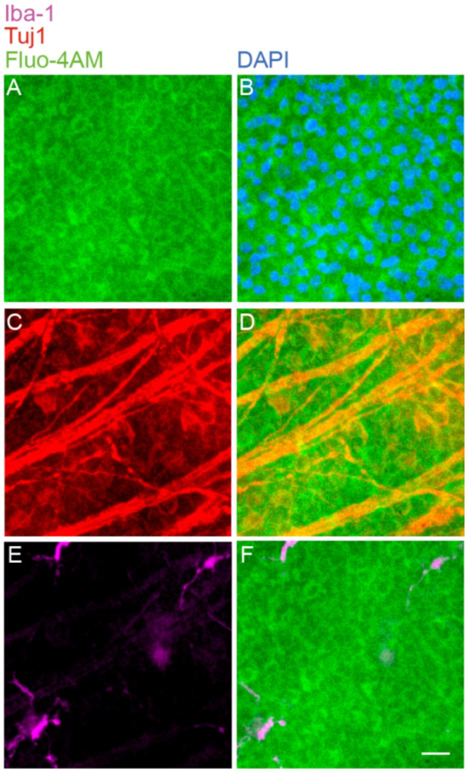

Figure S3. Immunofluorescent staining of retina after calcium imaging. Retina flatmount showing **(A)** calcium signal, **(B)** calcium signal merged with DAPI, **(C)** Tuj1 signal indicating RGCs and their axons, **(D)** calcium signal merged with Tuj1 signal, **(E)** Iba-1 signal indicating microglia and **(F)** Iba-1 signal merged with calcium signal. Scale bar indicates 50 μm.

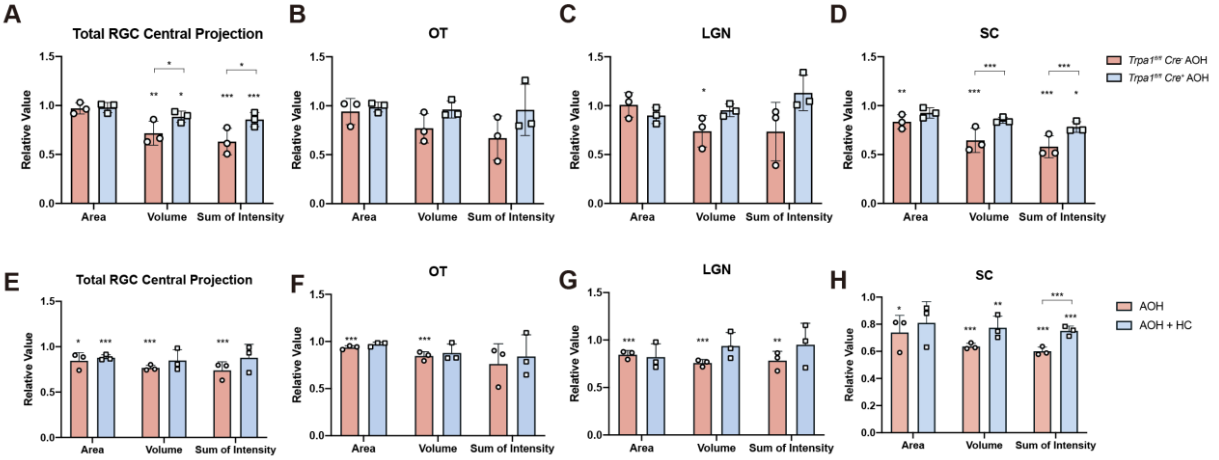

Figure S4. Histogram of RGC central projection changes under AOH with or without conditional knock-out of *Trpa1* in RGCs or HC treatment. (A) ~ (D) Histogram of RGC central projection changes under AOH with or without conditional knock-out of *Trpa1* in RGCs by animal crossbreeding in **(A)** whole RGC central projection; **(B)** optic tract; **(C)** LGN and **(D)** SC (n = 3 for each experiment, unpaired t-test, *P < 0.05, **P < 0.02, ***P < 0.01). **(E)** ~ **(H)** Histogram of RGC central projection changes under AOH with or without HC treatment in **(e)** whole RGC central projection; **(F)** optic tract; **(G)** LGN and **(H)** SC (n = 3 for each experiment, unpaired t-test, *P < 0.05, **P < 0.02, ***P < 0.01). Relative values were calculated as the value of AOH eyes divided by the corresponding contralateral eyes. Results are presented as mean ± standard deviation (SD). AOH, acute ocular hypertension; LGN, lateral geniculate nucleus; OT, optic tract; SC, superior colliculi.

Table S1.

Reagent, antibodies, equipment, and software.

REAGENT or RESOURCE	SOURCE	IDENTIFIER
**Antibodies**		
Rabbit anti-TRPA1	Invitrogen	Cat# PA1-46159; RRID: AB_2209939
Mouse anti-Tuj1	Abcam	Cat# ab78078; RRID: AB 2256751
Rabbit anti-CaMKII	Proteintech	Cat# 13731-1-AP
Rabbit anti-Phospho-CaMKII	CST	Cat# 12716
Rabbit anti-CREB1	Proteintech	Cat# 12208-1-AP
Rabbit anti-Phospho-CREB1	Proteintech	Cat# 28792-1-AP
p38	Abmart	Cat# T40071F
Phospho-p38	Abmart	Cat# T40076F
PE anti-mouse CD90.1 (Thy1.1)	BioLegend	Cat# 202523
Purified anti-mouse CD 16/32 antibody	BioLegend	Cat# 101301
Rabbit anti-Iba-1	MCE	Cat# HY-P80501
Donkey anti-Rabbit IgG (H+L) Secondary Antibody, Alexa Fluor™ 488	Thermo Fisher Scientific	Cat# A-21206
Donkey anti-Mouse IgG (H+L) Highly Cross-Adsorbed Secondary Antibody, Alexa Fluor™ 594	Thermo Fisher Scientific	Cat# A-21203
Donkey anti-Mouse IgG (H+L) Highly Cross-Adsorbed Secondary Antibody, Alexa Fluor™ 647	Thermo Fisher Scientific	Cat# A-31571
Anti-GAPDH antibody, HRP linked	Proteintech	Cat# HRP-60004
HRP goat-anti-rabbit secondary antibody	SAB	Cat# L3012
**Chemicals, peptides, and recombinant proteins**		
Cholera toxin B conjugated to Alexa Fluor 555	BrainVTA	Cat# CTB-02
Cholera toxin B conjugated to Alexa Fluor 488	BrainVTA	Cat# CTB-01
Fluo-4AM	Beyotime	Cat# S1060
HC-030031	Abcam	Cat# ab120554
Polygodial	Abcam	Cat# ab141518
KN-93	Selleck	Cat# S6787
Papain from papaya latex	Sigma	Cat# P4762
DNase I	Sigma	Cat# 10104159001
Cell staining buffer	BioLegend	Cat# 420201
Dichloromethane (DCM)	Sigma	Cat# 650463
Dibenzyl ester (DBE)	Sigma	Cat# 4526-74-3
**Critical commercial assays**		
In situ Cell Death Detection Kit	Roche	Cat# 12156792910
SteadyPure universal RNA extraction kit	Accurate Biology	Cat# AG21017
Primescript™ RT Master Takara Mix	Takara	Cat# RR036A
TB greenß premix ex taq	Takara	Cat# RR420A
BCA protein assay kit	Beyotime	Cat# P00105
Genotyping kit	Selleck	Cat# B40013
**Software and algorithms**		
Imaris	Oxford Instruments PLC, UK	RRID:SCR_007370
ImageJ	National Institute of Health, US	https://imagej.nih.gov/ij/; RRID:SCR_003070
Supermaze Video Tracking Software	Xinruan, Shanghai	https://www.shxinruan.com/dwxwfxrj/32.html
FlowJo	BD Biosciences	https://www.flowjo.com/solutions/flowjo/downloads/previous-versions
Amira	Visage Imaging	https://visageimaging.com

Graphpad Prism	GraphStats	https://www.graphpad.com/

Table S2.

Primer pairs applied in RT-qPCR.

Gene	Forward Sequence (5' to 3')	Reverse Sequence (5' to 3')
Trpc1	GATGTGCTTGGGAGAAATGCT	ACTGACAACCGTAGTCCAAAAG
Trpc2	CTCAAGGGTATGTTGAAGCAGT	GTTGTTTGGGCTTACCACACT
Trpc3	TCGAGAGGCCACACGACTA	GGTCACCTCCAGATGCTCATT
Trpc4	GTGTGCTACCTGATAGCTCCC	GGCAGAGACACGTTCGTTATT
Trpc5	GGGCTGAGACTGAGCTGTC	TTGCGGATGGCGTAGAGTAAT
Trpc6	AGCCAGGACTATTTGCTGATGG	AACCTTCTTCCCTTCTCACGA
Trpc7	CTTCCTGGACTCGGCTGAGTA	GCGTTCTGCCCCATGTAGT
Trpm1	CTGGGGCATGGTGGAGAAC	TGAGTGTGGGAATTGTTGAGC
Trpm2	CTTTGGGGTGCAGTCAAGGAG	TCCATGAGCTAAGGTTTTCTTGC
Trpm3	CAAGATGCCTGCCGTTTTTCT	GTCTAATTGGCAACTACCCCAAA
Trpm4	GGACTGCACACAGGCATTG	GTACCTTGCGGGGAATGAGC
Trpm5	CCAGCATAAGCGACAACATCT	GAGCATACAGTAGTTGGCCTG
Trpm6	TCTGCCACAATTTAGTCAGGTG	TGGTGCCGAAGGTATCTGTAG
Trpm7	AGGATGTCAGATTTGTCAGCAAC	CCTGGTTAAAGTGTTCACCCAA
Trpm8	ACAGACGTGTCCTACAGTGAC	GCTCTGGGCATAACCACACTT
Trpv1	CCGGCTTTTTGGGAAGGGT	GAGACAGGTAGGTCCATCCAC
Trpv2	TGCTGAGGTGAACAAAGGAAAG	TCAAACCGATTTGGGTCCTGT
Trpv3	ACGGTCACCAAGACCTCTC	GACTGTTGGGATTGGATGGGG
Trpv4	ATGGCAGATCCTGGTGATGG	GGAACTTCATACGCAGGTTTGG
Trpv5	ATGGGGGCTAAAACTCCTTGG	CCTCTTTGCCGGAAGTCACA
Trpv6	AGGGGTTAATACTCTGCCTATGG	GCACCTCACATCCTTCAAACTT
Trpa1	GTCCAGGGCGTTGTCTATCG	CGTGATGCAGAGGACAGAGAT
Piezo1	GCAGCCGAGAGACAGAGAAG	AGAGCAGAGGGAACCAGATGA

Piezo2	CAACGATGCAAGGACACACA	TCAAAGCCAATCTGCCGGAA
Gapdh	AGGTCGGTGTGAACGGATTTG	TGTAGACCATGTAGTTGAGGTCA

Table S3. The relative value of RGC central projections in different brain regions without or with RGC-specific knock out of Trpa1.

Region	*Parameter*	*$Trpa1^{fl/fl;}Cre^{-}$ (mean ± SD)*	*$Trpa1^{fl/fl};Cre^{+}$ (mean ± SD)*	*P value*
Total	Ar	0.971 ± 0.057	0.979 ± 0.051	0.911
	Vol	0.717 ± 0.123	0.885 ± 0.058	0.040
	SOI	0.631 ± 0.135	0.857 ± 0.032	0.009
OT	Ar	0.941 ± 0.133	0.986 ± 0.051	0.612
	Vol	0.770 ± 0.151	0.962 ± 0.088	0.129
	SOI	0.670 ± 0.226	0.958 ± 0.262	0.223
LGN	Ar	1.008 ± 0.125	0.900 ± 0.083	0.282
	Vol	0.738 ± 0.161	0.953 ± 0.064	0.099
	SOI	0.734 ± 0.100	1.131 ± 0.182	0.121
SC	Ar	0.835 ± 0.074	0.926 ± 0.053	0.157
	Vol	0.646 ± 0.125	0.851 ± 0.030	0.051
	SOI	0.580 ± 0.113	0.782 ± 0.055	0.049

Note: Each value (mean ± SD) represents the AOH side compared with the control side. OT: optic tract, OC: optic chiasm, LGN: lateral geniculate nuclei, SC: superior colliculi, Ar: area, Vol: volume, SOI: sum of intensity. $P < 0.05$ was recognized as statistically significant.
