# Supplementary material for: TRPA1 exacerbates selective retinal ganglion cell vulnerability under acute ocular hypertension
Source: Acta Neuropathol Commun. 2025 Apr 5;13:70. doi: 10.1186/s40478-025-01974-5 (PMC11971892; doi:10.1186/s40478-025-01974-5)
Supplement: Supplementary file 2 — Supplementary Material 2 [file 40478_2025_1974_MOESM2_ESM.pdf]

Supplementary File 2 for

TRPA1 exacerbates selective RGC vulnerability under acute ocular hypertension

Wenhan Lu, Yu Wang, Wei Hu, Xinyi Lin, Xiaoyu Tong, Yi Tian, Yuning Chen, Yicong Wang, Yan Xiao, Hongfang Yang*, Yi Feng*, Xinghuai Sun*

Correspondence to: xhsun@shmu.edu.cn (Xinghuai Sun)

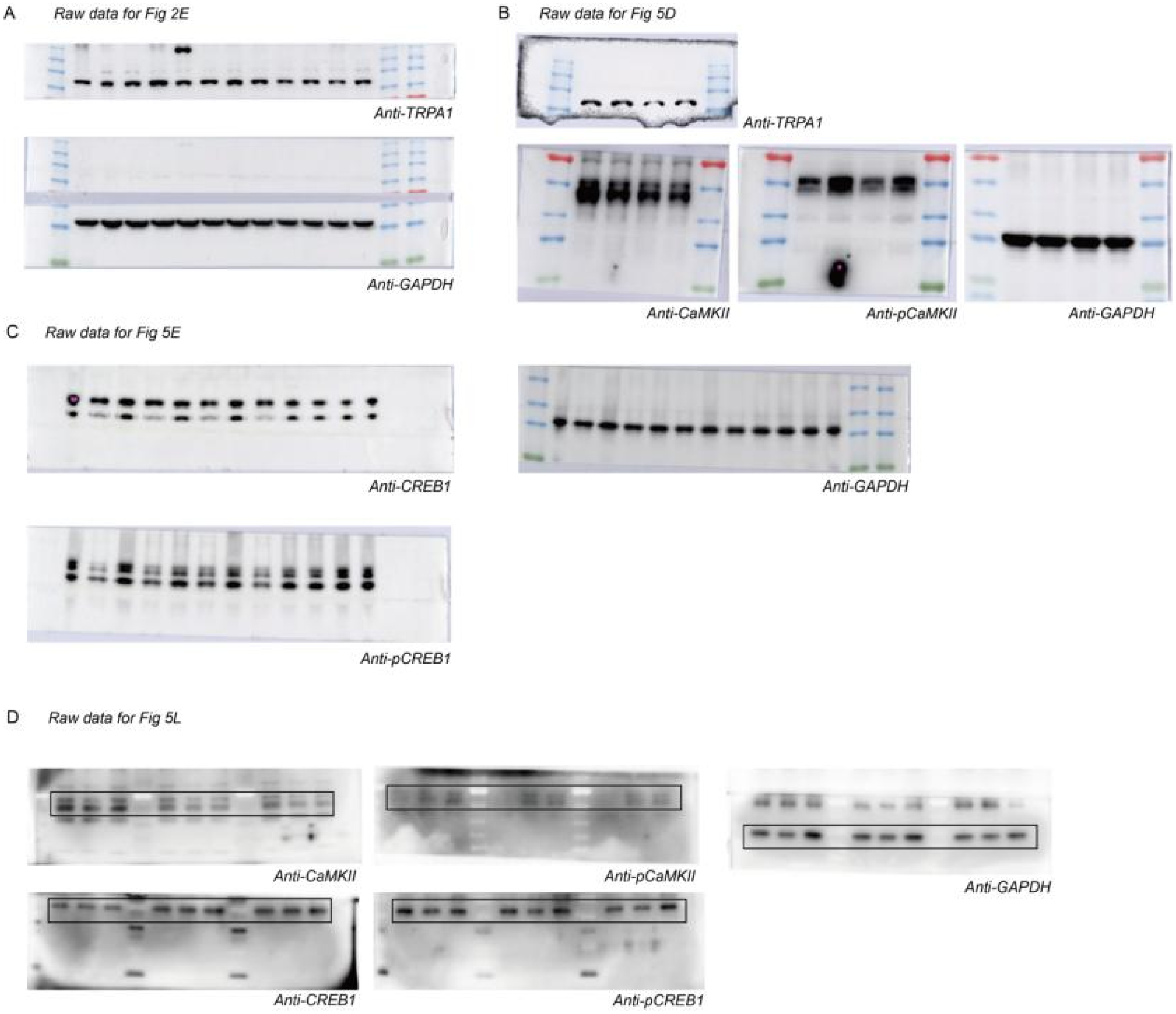

Original data of Western blot gels.
